# Kinase-mediated RAS signaling via membraneless cytoplasmic protein granules

**DOI:** 10.1016/j.cell.2021.03.031

**Published:** 2021-05-13

**Authors:** Asmin Tulpule, Juan Guan, Dana S. Neel, Hannah R. Allegakoen, Yone Phar Lin, David Brown, Yu-Ting Chou, Ann Heslin, Nilanjana Chatterjee, Shriya Perati, Shruti Menon, Tan A. Nguyen, Jayanta Debnath, Alejandro D. Ramirez, Xiaoyu Shi, Bin Yang, Siyu Feng, Suraj Makhija, Bo Huang, Trever G. Bivona

**Affiliations:** 1 Division of Pediatric Hematology/Oncology, UCSF, San Francisco, CA 94143, USA; 2 Department of Pharmaceutical Chemistry, UCSF, San Francisco, CA 94143, USA; 3 Department of Physics, University of Florida, Gainesville, FL 32611, USA; 4 Department of Medicine, Division of Hematology and Oncology, UCSF, San Francisco, CA 94143, USA; 5 Department of Pathology and Helen Diller Family Comprehensive Cancer Center, UCSF, San Francisco, CA 94143, USA; 6 UC Berkeley-UCSF Graduate Program in Bioengineering, UCSF, San Francisco, CA 94143, USA; 7 Department of Biochemistry and Biophysics, UCSF, San Francisco, CA 94143, USA; 8 Chan Zuckerberg Biohub, San Francisco, CA 94158, USA

**Keywords:** RAS, receptor tyrosine kinase, MAPK, kinase, ALK, anaplastic lymphoma kinase, RET, biomolecular condensate, protein granule, gene fusion

## Abstract

Receptor tyrosine kinase (RTK)-mediated activation of downstream effector pathways such as the RAS GTPase/MAP kinase (MAPK) signaling cascade is thought to occur exclusively from lipid membrane compartments in mammalian cells. Here, we uncover a membraneless, protein granule-based subcellular structure that can organize RTK/RAS/MAPK signaling in cancer. Chimeric (fusion) oncoproteins involving certain RTKs including ALK and RET undergo *de novo* higher-order assembly into membraneless cytoplasmic protein granules that actively signal. These pathogenic biomolecular condensates locally concentrate the RAS activating complex GRB2/SOS1 and activate RAS in a lipid membrane-independent manner. RTK protein granule formation is critical for oncogenic RAS/MAPK signaling output in these cells. We identify a set of protein granule components and establish structural rules that define the formation of membraneless protein granules by RTK oncoproteins. Our findings reveal membraneless, higher-order cytoplasmic protein assembly as a distinct subcellular platform for organizing oncogenic RTK and RAS signaling.

## Highlights

• RTK oncoproteins can form *de novo* membraneless cytoplasmic protein granules
• RTK protein granules activate RAS in a lipid membrane-independent manner
• Higher-order protein assembly is critical for oncogenic RAS/MAPK signaling
• Protein granules serve as a subcellular platform for organizing RTK signaling

## Graphical abstract

**Figure:**
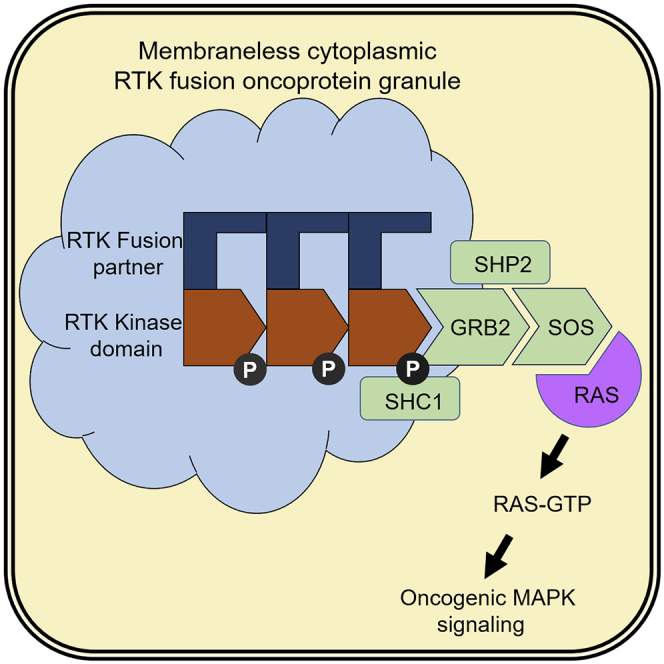

## Teaser

Receptor tyrosine kinase fusion oncoproteins undergo higher-order assembly to form membraneless cytoplasmic protein granules that coordinate local RAS activation and organize RAS/MAPK signaling in cancer

## Introduction

Receptor tyrosine kinase (RTK)/RAS/MAP kinase (MAPK) signaling is broadly important in regulating the proliferation and survival of normal human cells and is often hyper-activated through various mechanisms in human cancer (Sanchez-Vega et al., 2018). Native RTKs are integral membrane proteins, and canonical RTK signaling is thought to occur exclusively from lipid membrane subcellular compartments including the plasma membrane (PM) and intracellular organelles such as endosomes (Lemmon and Schlessinger, 2010). Moreover, RAS GTPase activation and downstream MAPK signaling is dependent upon the peripheral lipid membrane association of RAS proteins (Cox et al., 2015; Willumsen et al., 1984). Evidence of local RTK and RAS protein clustering in PM lipid-microdomains (Delos Santos et al., 2015; Plowman et al., 2005; Prior et al., 2003), and recent reports that the PM resident T cell receptor and associated proteins undergo phase separation in the presence of lipid bilayers, highlights the importance of physical compartmentalization of signaling events (Huang et al., 2019; Su et al., 2016). Distinct from the PM and lipid membrane-bound organelles, biomolecular condensates are an emerging mechanism of subcellular compartmentalization through primarily protein-based membraneless structures such as P-bodies, nucleoli, and stress granules (Alberti et al., 2019; Shin and Brangwynne, 2017). Although connections between aberrant transcription factor condensates and cancer have been proposed (Boulay et al., 2017; Koken et al., 1994), the functional role of biomolecular condensates in oncogenic signaling and cancer pathogenesis remains to be defined.

Prominent examples of oncogenic RTK/RAS/MAPK signaling in cancer include naturally occurring chromosomal rearrangements involving RTKs such as anaplastic lymphoma kinase (ALK) or rearranged during transfection (RET), which generate chimeric (fusion) oncoproteins that are validated therapeutic targets across multiple cancer subtypes (Childress et al., 2018; Kato et al., 2017). Virtually all oncogenic ALK and RET fusion proteins retain the intracellular domain, which includes the kinase, but lack the native transmembrane domain (Childress et al., 2018; Kato et al., 2017). The absence of a canonical lipid membrane targeting domain as a shared structural feature of many oncogenic RTK fusion proteins presents fundamental cell biological questions (Nelson et al., 2017): (1) Where and how do these RTK oncoproteins organize signaling in cells? and (2) How do these RTK fusion oncoproteins activate RAS signaling, which is thought to occur exclusively on lipid membrane compartments in mammalian cells?

We previously discovered that the echinoderm microtubule-associated protein-like 4 (EML4)-ALK fusion oncoprotein that is present recurrently in lung cancer and other cancer subtypes is exquisitely dependent upon RAS GTPase activation and downstream RAF/MEK/ERK (MAPK pathway) signaling for its oncogenic output (Hrustanovic et al., 2015). We and other groups showed that EML4-ALK is not localized to the PM, but instead to intracellular, punctate cytoplasmic structures of unknown identity (Hrustanovic et al., 2015; Richards et al., 2015). This specific intracellular localization is essential for EML4-ALK to activate RAS and downstream MAPK signaling (Hrustanovic et al., 2015). Neither the cell biological, biophysical, or biochemical nature of these cytoplasmic structures nor the mechanism through which they promote oncogenic RTK and RAS signaling is clear. In this study, we uncover a previously unrecognized platform for RTK/RAS/MAPK signaling in mammalian cells: membraneless cytoplasmic protein granules.

## Results

### EML4-ALK forms *de novo* membraneless cytoplasmic protein granules

**Figure 1:**
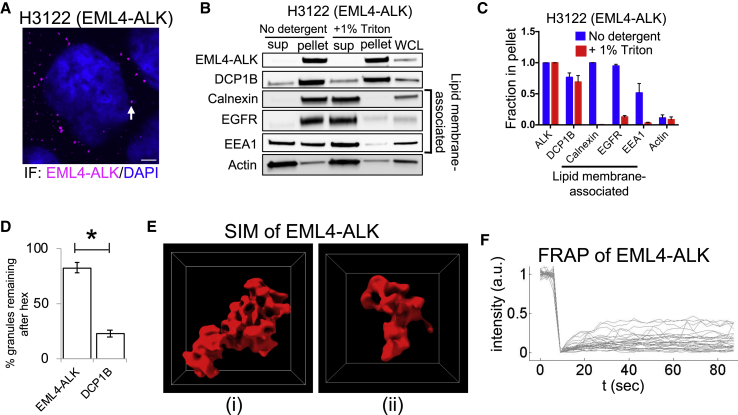
EML4-ALK forms *de novo* membraneless cytoplasmic protein granules (A) Anti-ALK IF in H3122 cells representative of 20+ cells, n = 3. Arrow indicates a representative EML4-ALK puncta. Scale bar, 5 μm. (B and C) Subcellular fractionation ±1% Triton X-100 in H3122 cells, followed by western blotting (B). EML4-ALK and DCP1B are statistically distinct (p < 0.05, one-way ANOVA with post hoc Tukey’s HSD test) from the lipid membrane-associated proteins, which shift from the insoluble fraction (pellet) to the supernatant (sup) with detergent. n = 3. (D) EML4-ALK or DCP1B granule persistence after 5 min of 5% hexanediol (hex) treatment. Error bars represent ±SEM, ^∗^p value < 0.05 by unpaired t test. (E) SIM images of 2 distinct YFP::EML4-ALK puncta in Beas2B cells. SIM box: 2 μm^3^. (F) FRAP analysis of YFP::EML4-ALK puncta in Beas2B cells. N = 30 cells. See also Figure S1 and Video S1.

**Figure S1:**
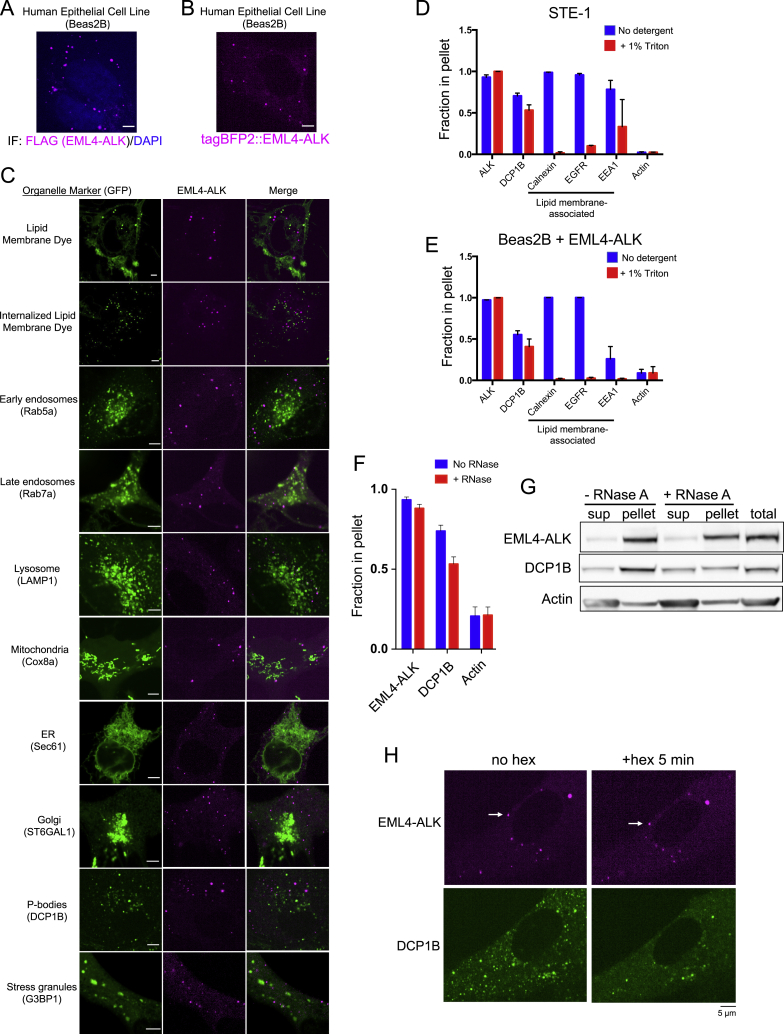
Cell biological and biophysical properties of EML4-ALK membraneless cytoplasmic protein granules, related to Figure 1 (A) Anti-FLAG immunofluorescence of FLAG-tagged EML4-ALK expressed in human epithelial cell line Beas2B. DAPI serves as a nuclear stain. Image is representative of at least 75 analyzed cells in total over 3 independent experiments. Scale bar = 5 μM. (B) Live-cell confocal imaging of human epithelial cell line Beas2B upon expression of mTagBFP2::EML4-ALK. Image is representative of 200 analyzed cells in total over 5 independent experiments. Scale bar = 5 μM. (C) Live-cell confocal imaging of human epithelial cell line Beas2B upon expression of mTagBFP2::EML4-ALK and mEGFP-tagged organelle markers as listed. Membrane dye experiments were conducted using live cells incubated with CellTracker CM-DiI Dye (Invitrogen). Each panel is a representative image of at least 20 analyzed cells per condition in 3 independent experiments. Scale bar = 5 μM. (D, E) Subcellular fractionation by ultracentrifugation ± detergent (1% Triton X-100) to disrupt lipid membranes in STE-1 (D), an EML4-ALK expressing cancer cell line, and Beas2B cells expressing EML4-ALK (E). In both cell lines, EML4-ALK and DCP1B are statistically distinct from the lipid membrane-associated proteins, which shift from the insoluble fraction (pellet) to the soluble fraction with detergent (p < 0.05 for all comparisons by one-way ANOVA with post hoc Tukey’s HSD test, except for DCP1B versus EEA1 in panel D and E and ALK versus EEA1 in panel E). Bar graphs reflect quantification of western blotting results for 3 independent replicates. Fraction in pellet calculated as ratio of the insoluble fraction to total (insoluble plus supernatant fractions) as assessed by western blotting. Error bars represent ± SEM. (F, G) Subcellular fractionation by ultracentrifugation ± RNase A treatment for 30 minutes in EML4-ALK expressing cancer cell line H3122. P-body protein DCP1B partially shifts from the insoluble (pellet) fraction to the supernatant (sup) upon RNase A treatment, in contrast to EML4-ALK. Western blotting images are representative of at least 5 independent experiments. Fraction in pellet (G) calculated as ratio of the insoluble fraction to total (insoluble plus supernatant fractions) as assessed by western blotting (F). DCP1B demonstrates a significant RNase-dependent reduction in the insoluble fraction (p < 0.05 by paired t test) compared to EML4-ALK. (H) Live-cell imaging of human epithelial cell line Beas2B co-expressing mTagBFP2::EML4-ALK and eGFP::DCP1B treated with 5% hexanediol (hex) and imaged at respective time points. Images are representative of at least 5 analyzed cells in 3 independent experiments. White arrows indicate a representative EML4-ALK cytoplasmic protein granule (multiple non-highlighted granules are also pictured in all panels). Quantification of granule persistence shown in Main Figure 1D.

We set out to identify the cytoplasmic structure to which ALK fusion oncoproteins localize in cells. We focused our initial study on EML4-ALK variant 1, the most common oncogenic form in human cancers (Sabir et al., 2017). First, we confirmed that EML4-ALK localized to punctate structures in the cytoplasm, and not to the PM, by immunofluorescence (IF) in patient-derived cancer cells (H3122) that endogenously express this EML4-ALK variant (Hrustanovic et al., 2015) (Figure 1A). We validated the similar presence of EML4-ALK cytoplasmic puncta upon expression in a non-transformed human bronchial epithelial cell line (Beas2B), both by IF analysis in Beas2B cells expressing FLAG-tagged EML4-ALK (Figure S1A) and by live-cell imaging in Beas2B cells expressing fluorescent protein-tagged EML4-ALK (Figure S1B). These imaging results confirm localization of EML4-ALK at cytoplasmic puncta and indicate these subcellular structures are not the result of artificial expression, fixation, or fluorescent protein-mediated multimerization (Cranfill et al., 2016).

We next tested whether EML4-ALK cytoplasmic puncta correspond to an intracellular lipid membrane-containing structure, given the well-established role of lipid membranes in organizing RTK signaling and the requirement of lipid membranes for RAS GTPase activation (Delos Santos et al., 2015; Jackson et al., 1990; Willumsen et al., 1984). Live-cell imaging in Beas2B cells showed no significant colocalization of EML4-ALK cytoplasmic puncta with the PM or intracellular membranes as marked by a lipid membrane intercalating dye or with a panel of established protein markers labeling canonical intracellular lipid-containing organelles (Rizzuto et al., 1995) (Figure S1C). To further evaluate whether EML4-ALK associates with lipid membranes, we performed subcellular fractionation in patient-derived cancer cell lines expressing endogenous EML4-ALK. EML4-ALK displayed a fractionation pattern unaffected by membrane-solubilizing detergents, which was distinct from the pattern of PM-spanning (epidermal growth factor receptor [EGFR]) or internal membrane proteins (calnexin and early endosome antigen 1 [EEA1]), yet similar to that of a well-known cytoplasmic ribonucleoprotein granule constituent (the P-body protein de-capping mRNA 1B, DCP1B) (Aizer et al., 2008) (Figures 1B, 1C, S1D, and S1E). These findings indicated that EML4-ALK may exist in a membraneless subcellular compartment within the cytoplasm. We confirmed that EML4-ALK puncta do not colocalize with the two known biomolecular condensates in the cytoplasm, P-bodies and stress granules (Figure S1C). Moreover, EML4-ALK puncta are not disrupted by RNase A, in contrast to ribonucleoprotein granules like the P-body (Figures S1F and S1G). These results suggest that EML4-ALK forms distinct protein-based, instead of RNA-protein-based, membraneless cytoplasmic granules.

We then investigated the biophysical properties of EML4-ALK using a suite of established cellular assays for characterizing biomolecular condensates (Molliex et al., 2015; Patel et al., 2015). We found that EML4-ALK granules are not simple, homogeneous, liquid droplets. No fission or fusion events were observed during the time window of our live cell imaging in spite of occasional granule collisions (Video S1) (Alberti et al., 2019). Most granules persist after hexanediol treatment, unlike liquid-like P-bodies labeled by DCP1B (Figures 1D and S1H) (Kroschwald et al., 2015). EML4-ALK granules exhibit porous and curvilinear shapes as revealed by super-resolution structured illumination microscopy (SIM) (Figure 1E), instead of the more smooth and spherical appearance characteristic of liquid droplets (Patel et al., 2015; Shin and Brangwynne, 2017). Fluorescence recovery after photo-bleaching (FRAP) showed heterogeneous EML4-ALK exchange between the granules and the surrounding cytosol among granules (Figure 1F), with a median recovery of ∼10% at 1 min and some granules showing up to 40% recovery (Figure 1F). Taken together, our results indicate EML4-ALK forms *de novo* cytoplasmic protein granules that exhibit heterogeneity across a continuum of biophysical states, with solid-like properties more prevalent than liquid-like ones. The heterogeneity in biophysical state may reflect an ongoing liquid-to-solid maturation and aging process of the granules (Jain et al., 2016; Molliex et al., 2015).

Video S1. EML4-ALK granule live-cell microscopy, related to Figure 1 Live-cell confocal microscopy video of Beas2B cells expressing mTagBFP2::EML4-ALK showing EML4-ALK granules in direct contact without any evidence of fusion or fission events.

### EML4-ALK membraneless cytoplasmic protein granules recruit the RAS-activating complex GRB2/SOS1/GAB1 *in situ*

**Figure 2:**
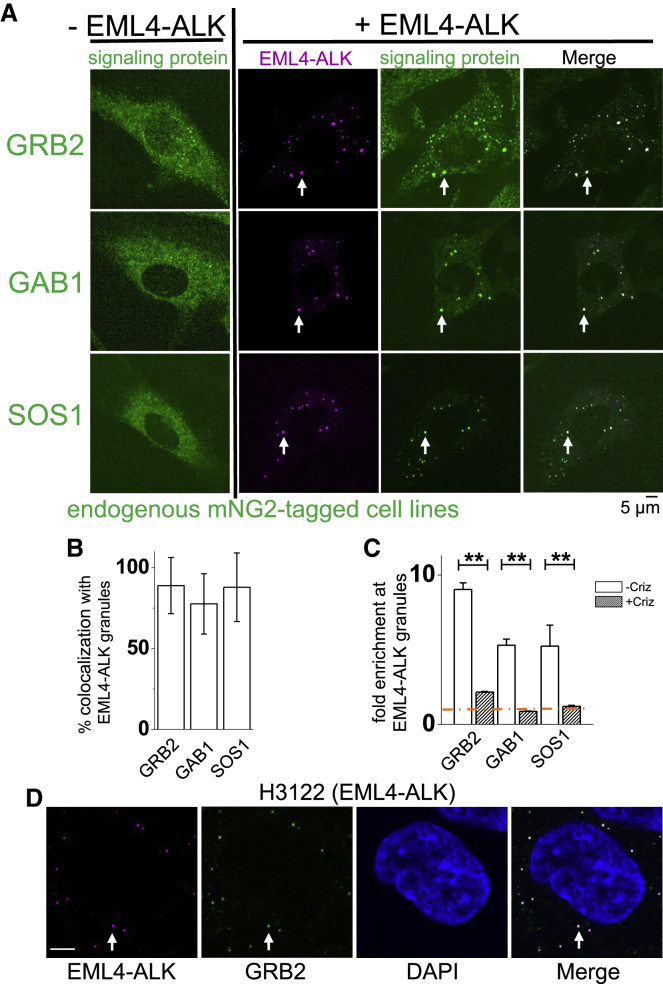
EML4-ALK membraneless cytoplasmic protein granules recruit RAS-activating complex GRB2/SOS1/GAB1 *in situ* (A) Live-cell imaging of Beas2B cells with endogenous mNG2-tagging of signaling proteins ± mTagBFP2::EML4-ALK. (B) Signaling protein colocalization with EML4-ALK granules. 100 total cells per condition n = 3. (C) Fold-enrichment of signaling proteins at EML4-ALK granules ±24 h treatment with 1 μM crizotinib (criz). ^∗∗^p < 0.01, paired t test. Orange line denotes zero enrichment. (D) Anti-ALK IF images in H3122 cells with endogenous mNG2-tagging of GRB2 representative of 20+ cells, n = 3. Scale bar, 5 μM. For (A) and (D), arrows indicate a representative EML4-ALK granule with local enrichment of signaling proteins (multiple non-highlighted granules also show colocalization). For all panels, error bars represent ±SEM. See also Figure S2.

**Figure S2:**
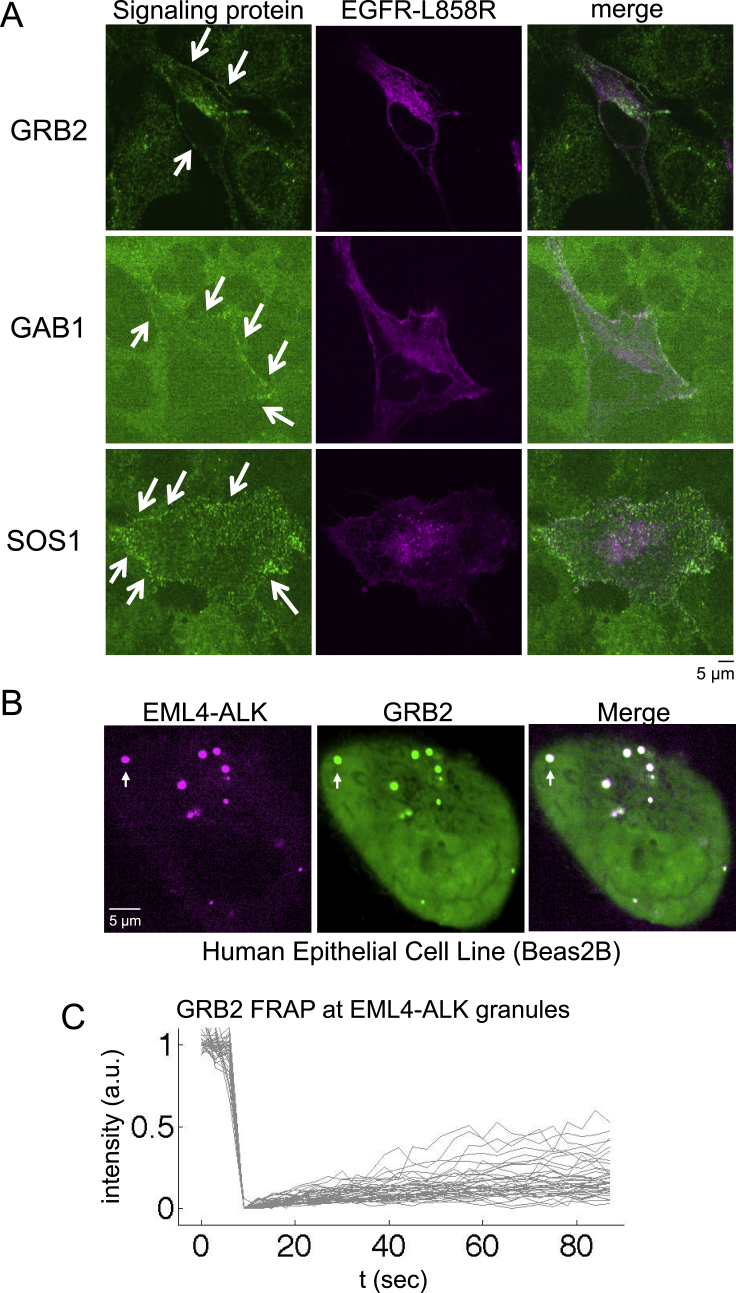
RAS adaptor protein GRB2 is recruited to EML4-ALK cytoplasmic protein granules, related to Figure 2 (A) Live-cell confocal imaging of mTagBFP2::EGFR L858R (oncogenic EGFR) expressed in human epithelial cell lines (Beas2B) with endogenous mNeonGreen2-tagging of GRB2, GAB1, and SOS1. Arrows denote plasma membrane enrichment of GRB2, GAB1, and SOS1. Representative images from at least 30 cells analyzed per condition in 3 independent experiments. (B) Live-cell confocal imaging of mTagBFP2::EML4-ALK and mEGFP::GRB2 upon dual expression in Beas2B cells. White arrows indicate a representative EML4-ALK cytoplasmic protein granule with local enrichment of GRB2 (multiple non-highlighted granules also show colocalization between EML4-ALK and GRB2). Images are representative of 100 analyzed cells in total over 3 independent experiments. (C) FRAP experiments performed in human epithelial cells (Beas2B) with endogenous mNeonGreen2-tagging of GRB2, upon expression of mTagBFP2::EML4-ALK. Graph displays individual recovery of fluorescence intensity after photobleaching of GRB2 enriched at EML4-ALK granules, t denotes time in seconds, intensity in arbitrary units (a.u.) normalized to 1. N = 30 cells.

To uncover the connection between EML4-ALK membraneless cytoplasmic granules and RAS activation, we created a library of gene-edited Beas2B cell lines by introducing a split mNeonGreen2_1–10/11_ tag (mNG2) at the endogenous locus of canonical adaptor and effector proteins in the RTK/RAS/MAPK signaling pathway, including GRB2, GAB1, SOS1, and RAS GTPases (H/N/K isoforms) (Feng et al., 2017). This suite of isogenic cell lines avoids potential biases that can arise when overexpressing labeled proteins or fixing and permeabilizing cells for immunofluorescence. In this set of cell lines, we found that expression of EML4-ALK specifically re-localized key upstream RAS pathway proteins, including GRB2, GAB1, and SOS1, from a mainly cytosolic pattern to the discrete EML4-ALK granules and not to the PM (Figures 2A and 2B). This is distinct from the pattern of PM re-localization seen in the control case of expressing an oncogenic form of the transmembrane RTK EGFR (Figure S2A). Treatment with the ALK kinase inhibitor crizotinib for 24 h substantially reduced the recruitment of these adaptor proteins without affecting cell viability, indicating that this process requires ALK kinase activation (Figure 2C). We orthogonally confirmed recruitment of the key adaptor, GRB2, to endogenous EML4-ALK cytoplasmic protein granules detected by IF in patient-derived cancer cells (H3122) (Figure 2D), as well as through dual expression of EML4-ALK and GRB2 in Beas2B cells (Figure S2B). Additionally, we observed a low and heterogeneous FRAP recovery behavior for GRB2 at the EML4-ALK protein granules, similar to that of EML4-ALK itself (Figure S2C).

### Cytoplasmic EML4-ALK protein granules locally activate RAS

**Figure S3:**
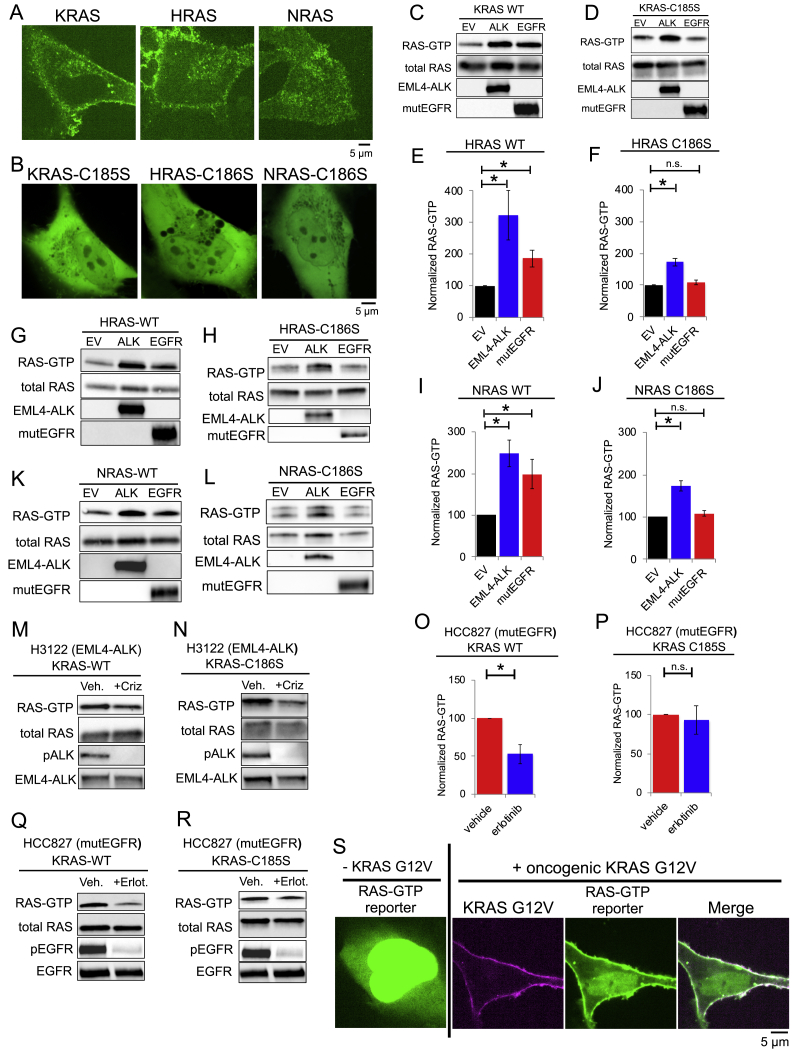
EML4-ALK-specific activation of cytosolic RAS, related to Figure 3 (A) Live-cell confocal imaging of human epithelial cell lines (Beas2B) with endogenous mNeonGreen2-tagging of KRAS, HRAS, and NRAS. Images are representative of at least 20 analyzed cells per condition in 3 independent experiments. (B) Live-cell confocal imaging of human epithelial cell line Beas2B expressing mEGFP-tagged cytosolic RAS mutants (KRAS-C185S, HRAS-C186S, NRAS-C186S). Images are representative of at least 25 analyzed cells per condition in 3 independent experiments. (C, D) KRAS wild-type (WT) or cytosolic KRAS-C185S were stably expressed in 293T cells and then transfected with either empty vector (EV), EML4-ALK, or oncogenic EGFR-L858R (mutEGFR). Western blotting images are representative of at least 3 independent experiments and RAS-GTP levels are quantified in Main Figures 3A and 3B. (E-L) HRAS/NRAS wild-type or cytosolic mutants (HRAS C186S, NRAS C186S) were stably expressed in 293T cells and then transfected with either empty vector (EV), EML4-ALK, or oncogenic EGFR-L858R (mutEGFR). RAS-GTP levels were normalized to the relevant total RAS protein level (H/NRAS wild-type or C186S). n = 4. Error bars represent ± SEM, ^∗^ denotes p value < 0.05, n.s. denotes non-significant comparison, one-way ANOVA with post hoc Tukey’s HSD test. Western blotting images are representative of 4 independent experiments. (M, N) EML4-ALK expressing H3122 cancer cell line with stable expression of KRAS wild-type or cytosolic KRAS C185S mutant ± 2 hours of 100 nM crizotinib. Western blotting images are representative of 3 independent experiments and RAS-GTP levels are quantified in Main Figures 3C and 3D. (O-R) Patient-derived oncogenic EGFR expressing cell line (HCC827) with stable expression of either wild-type KRAS or cytosolic mutant (KRAS C185S). RAS-GTP levels determined ± 2 hours of 100 nM erlotinib treatment and normalized to the relevant total RAS protein level (KRAS wild-type or C185S). n = 4. Error bars represent ± SEM, ^∗^ denotes p value < 0.05, n.s. denotes non-significant comparison, paired t test. Western blotting images are representative of 4 independent experiments. (S) Live-cell confocal imaging of human epithelial cell line Beas2B with GFP-labeled RAS-GTP reporter (tandem GFP-RBD). Left column demonstrates baseline RAS-GTP reporter localization to cytosol and nucleoplasm, right three panels show plasma membrane re-localization of RAS-GTP reporter upon expression of mTagBFP2::KRAS G12V (oncogenic KRAS). Images are representative of 15-25 cells analyzed per condition in 3 independent experiments.

**Figure 3:**
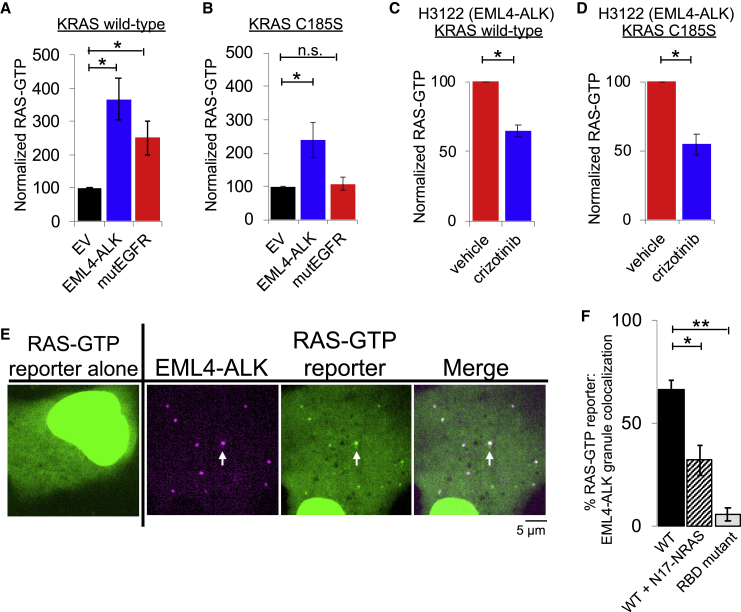
Cytoplasmic EML4-ALK protein granules locally activate RAS (A–D) RAS-GTP levels of KRAS wild-type (A and C) or C185S cytosolic mutant (B and D) stably expressed in 293T or H3122 cells, followed by transfection of empty vector (EV), EML4-ALK, or oncogenic EGFR (A and B) or ±2 h of 100 nM crizotinib (C and D). n = 3. See STAR Methods for normalization details. (E) Live-cell imaging of RAS-GTP reporter in Beas2B cells ± mTagBFP2::EML4-ALK. Arrow indicates a representative EML4-ALK granule with local enrichment of RAS-GTP (multiple non-highlighted granules also show colocalization). (F) Per cell quantification of EML4-ALK granule/RAS-GTP reporter colocalization. WT denotes unmodified GFP-RBD reporter, RBD mutant (R59A/N64D) displays diminished RAS-GTP binding. n = 3, 30+ cells per replicate. For all panels, error bars represent ± SEM, ^∗^p < 0.05, ^∗∗^p < 0.01, n.s., non-significant comparison, one-way ANOVA with post hoc Tukey’s HSD test (A, B, and F) or paired t test (C and D). See also Figure S3.

Our imaging and biochemical data prompted the unanticipated hypothesis that RTK-mediated RAS GTPase activation may occur via a subcellular structure lacking lipid membranes (i.e., EML4-ALK membraneless cytoplasmic protein granules), potentially through a cytosolic pool of RAS that is known to exist but with unclear functional significance (Goodwin et al., 2005; Zhou et al., 2016). We first confirmed RAS protein expression in the cytosol, in addition to lipid membrane subcellular compartments (Figure S3A). Next, we directly tested whether cytosolic RAS could become activated in a lipid membrane-independent manner by EML4-ALK cytoplasmic protein granules. We utilized established mutant forms of RAS (KRAS-C185S, H/NRAS-C186S) that abrogate lipid membrane targeting and are retained exclusively in the cytosol (Jackson et al., 1990) (Figure S3B). Whereas the expression of either EML4-ALK or the PM-localized oncogenic EGFR increased wild-type RAS-GTP levels (Figures 3A, S3C, S3E, S3G, S3I, and S3K), only EML4-ALK increased RAS-GTP levels of cytosolic RAS mutants (Figures 3B, S3D, S3F, S3H, S3J, and S3L). Furthermore, inhibition of EML4-ALK with crizotinib in H3122 patient-derived cancer cells suppressed not only wild-type RAS-GTP levels, but also the levels of GTP-bound, cytosolic KRAS-C185S (Figures 3C, 3D, S3M, and S3N). Control experiments treating a distinct patient-derived cancer cell line HCC827 expressing endogenous oncogenic EGFR (PM-localized) with an established EGFR inhibitor (Tsao et al., 2005) confirmed suppression of wild-type RAS-GTP levels but showed no effect on KRAS-C185S RAS-GTP levels (Figures S3O–S3R). These findings demonstrate the specificity of cytosolic RAS activation by oncogenic EML4-ALK.

Last, to determine whether EML4-ALK cytoplasmic granules display evidence of local RAS activation (i.e., RAS-GTP), we used an established tandem GFP-RBD (RAS-binding domain) live-cell reporter given its high-affinity binding to RAS-GTP and sensitivity for detection of endogenous RAS activation (Biskup and Rubio, 2014). When expressed alone, the RAS-GTP reporter displayed homogeneous localization in the cytosol and enrichment in the nucleoplasm, as previously described (Rubio et al., 2010) (Figure 3E). As a positive control, expression of oncogenic KRAS in Beas2B cells led to re-localization of the RAS-GTP reporter to the PM (Figure S3S). In EML4-ALK-expressing cells, we observed substantial enrichment of the RAS-GTP reporter at EML4-ALK cytoplasmic protein granules and not at the PM (Figures 3E and 3F). Co-expression of a dominant-negative RAS (RASN17) (Sigal et al., 1986) that interferes with RAS activation (GTP-loading) decreased colocalization of the RAS-GTP reporter at EML4-ALK granules, as did introduction of mutations into the RBD component of the GFP-RBD reporter (RBD R59A/N64D) that decrease affinity for RAS-GTP (Biskup and Rubio, 2014) (Figure 3F). The collective findings show that local RAS activation and accumulation of RAS-GTP occurs at membraneless EML4-ALK cytoplasmic protein granules.

### Protein granule formation by EML4-ALK is critical for RAS/MAPK signaling

**Figure 4:**
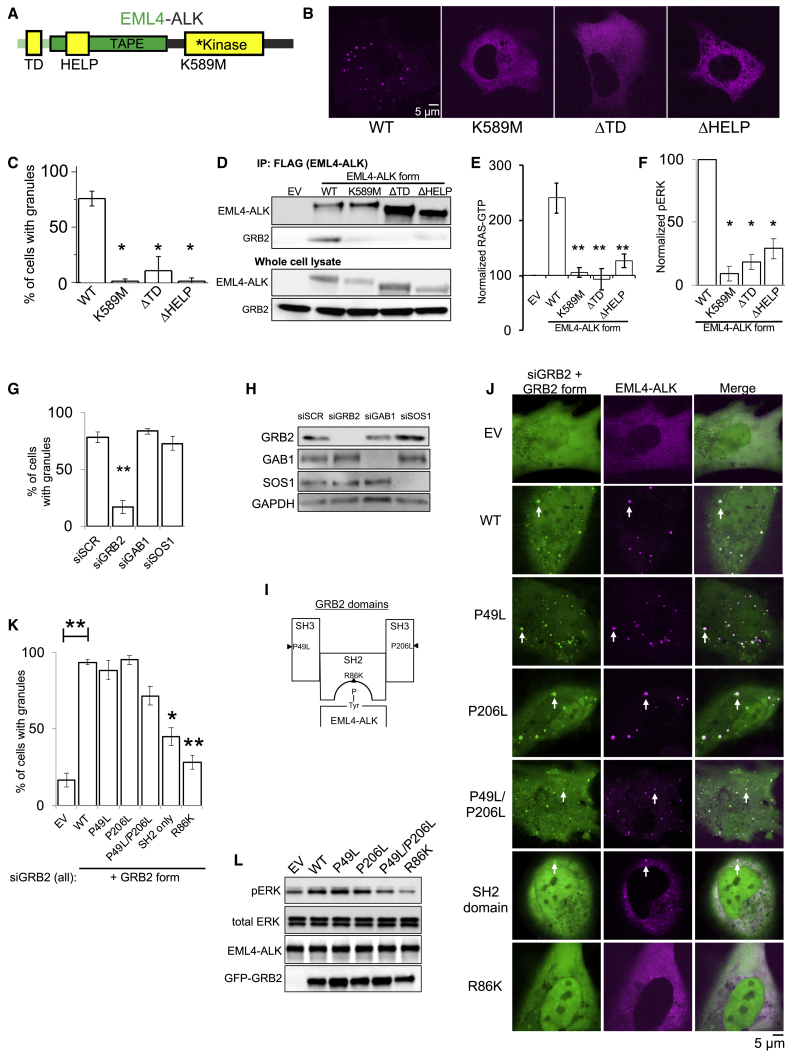
Protein granule formation by EML4-ALK is critical for RAS/MAPK signaling (A) Domain structure schematic of EML4-ALK. (B) Live-cell imaging of mTagBFP2::EML4-ALK wild-type (WT) or mutant forms in Beas2B cells. (C) Quantification of % cells with granules (6 or greater). 75 cells per condition, n = 3. (D) Anti-FLAG co-IP of FLAG-tagged WT or mutant EML4-ALK forms in 293T cells, followed by GRB2 western blotting. n = 3. (E and F) Endogenous RAS-GTP levels (E) and ERK activation (F) by western blotting upon expression of EML4-ALK WT or mutant forms in 293T cells. n = 4. See STAR Methods for normalization details. (G and H) Live-cell imaging of mTagBFP2::EML4-ALK in Beas2B cells after small interfering RNA (siRNA) treatment (72 h). Quantification of % cells with granules, 100 total cells, n = 3 (G). Representative images in Figure S4D. Western blotting for siRNA knockdown, n = 3 (H). (I) Structure schematic of adaptor protein GRB2. (J) Live-cell imaging of mTagBFP2::EML4-ALK and mEGFP-labeled GRB2 mutants in Beas2B cells after 72 h siRNA against endogenous GRB2. SH2 only denotes the GRB2 SH2 domain. Arrows indicate representative EML4-ALK granules with local enrichment of GRB2 mutants (multiple non-highlighted granules also show colocalization). (K) Quantification of % cells with EML4-ALK granules (6 or greater). 75 total cells per condition, n = 3. (L) Western blotting upon co-expression of EML4-ALK and mEGFP-labeled GRB2 mutants in 293T cells after 72 h of siRNA against endogenous GRB2. For all panels, error bars represent ±SEM, ^∗^p < 0.05, ^∗∗^p < 0.01, one-way ANOVA with post hoc Tukey’s HSD test.

**Figure S4:**
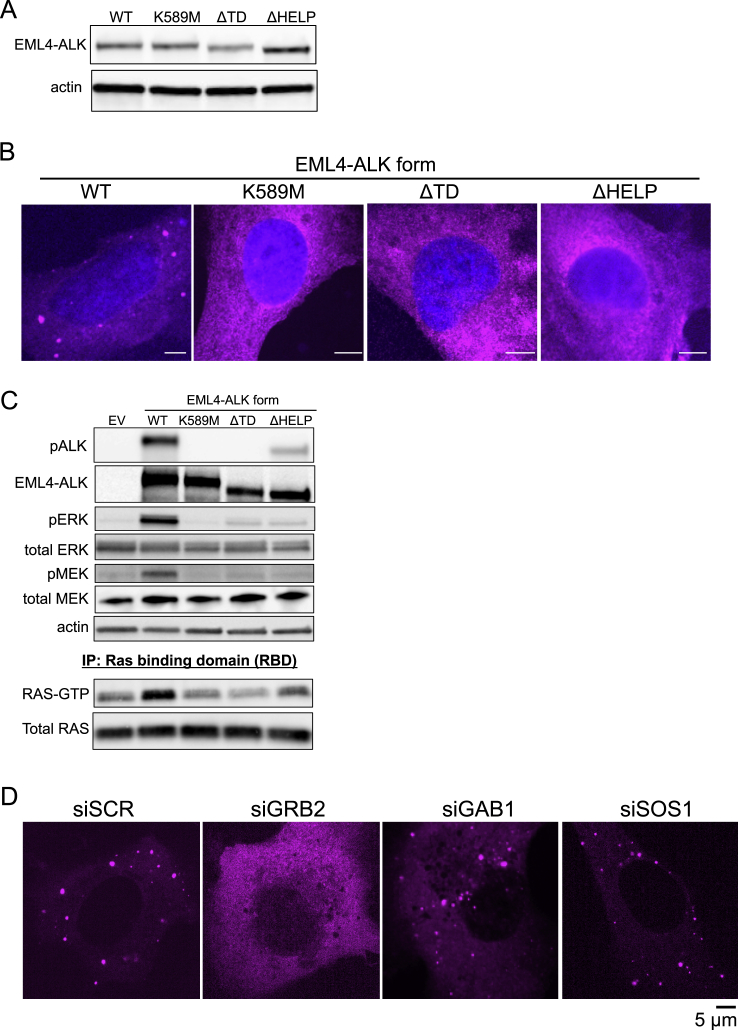
Non-granule-forming mutants of EML4-ALK disrupt RAS/MAPK signaling, related to Figure 4 (A) Western blotting upon expression of wild-type EML4-ALK or EML4-ALK kinase-deficient (K589M), ΔTD or ΔHELP mutants in human epithelial cell line Beas2B. Representative images and quantification of percentage of cells with granules shown in Main Figures 4B and 4C. (B) Anti-FLAG immunofluorescence of human epithelial cell line Beas2B expressing either FLAG-tagged EML4-ALK wild-type (WT) or EML4-ALK kinase-deficient (K589M), ΔTD or ΔHELP mutants. EML4-ALK (FLAG) staining in pink, DAPI in blue. Images are representative of at least 35 cells analyzed per condition in 2 independent replicates. Scale bar = 5 μM. (C) Western blotting and immunoprecipitation (IP) for levels of endogenous RAS activation (GTP-bound RAS) upon expression of wild-type EML4-ALK or EML4-ALK kinase-deficient (K589M), ΔTD or ΔHELP mutants in 293T cells. Images are representative of at least 5 independent experiments. RAS-GTP and pERK levels are quantified in Main Figures 4E and 4F. (D) Live-cell confocal imaging upon expression of mTagBFP2::EML4-ALK in Beas2B cells after 72 hours of siRNA treatment. Images are representative of at least 4 independent experiments and percent granule formation is quantified in Main Figure 4G.

We next tested whether downstream MAPK signaling output is dependent on EML4-ALK cytoplasmic protein granules by investigating the molecular determinants of *de novo* granule formation. The EML4 portion of the chimeric EML4-ALK oncoprotein contains an N-terminal trimerization domain (TD) and a truncated tandem atypical WD-propeller in EML4 protein (TAPE) domain (Sabir et al., 2017) (Figure 4A). Deletion of the TD or the hydrophobic EML protein (HELP) motif in the TAPE domain disrupted protein granule formation resulting, instead, in a diffuse cytosolic distribution of EML4-ALK labeled by either fluorescent protein or FLAG-tag (Figures 4B, 4C, S4A, and S4B). ΔTD or ΔHELP mutants of EML4-ALK demonstrated loss of ALK *trans*-phosphorylation and GRB2 interaction (Figures 4D and S4C) and impaired RAS/MAPK activation (Figures 4E, 4F, and S4C). These data implicate *de novo* protein granule formation that is mediated by the EML4 portion of the fusion protein as critical for productive RAS/MAPK signaling. An established kinase-deficient mutant (K589M) form of EML4-ALK abrogated RAS/MAPK signaling and unexpectedly also disrupted protein granule formation (Figures 4B–4F, S4B, and S4C).

To investigate why EML4-ALK protein granule assembly required the kinase activity of EML4-ALK, we hypothesized that ALK kinase transphosphorylation enabled phospho-site docking of adaptor proteins that could contribute to condensate formation, potentially via multivalent protein interactions (Su et al., 2016). We tested whether key protein components that link RTK activation to RAS signaling were required for EML4-ALK cytoplasmic granule formation. Knockdown of GRB2, but not of SOS1 or GAB1, significantly impaired EML4-ALK granule formation (Figures 4G and 4H). Using established domain-specific mutants of GRB2 (Figure 4I) (Cussac et al., 1994; Lowenstein et al., 1992), we observed that an SH2 domain mutant of GRB2 (R86K) deficient in phospho-tyrosine binding was unable to rescue EML4-ALK granule formation in cells with endogenous GRB2 depleted, unlike expression of wild-type GRB2 (Figures 4J and 4K). This demonstrates the importance of kinase-dependent GRB2 phospho-site docking onto activated EML4-ALK for granule formation.

We also tested the GRB2 SH2 domain alone and single (P49L and P206L) or double (P49L/P206L) SH3 mutant forms of GRB2 with diminished SH3-dependent binding to adaptor and effector proteins (e.g., SOS1, GAB1) but proficient for SH2-dependent phospho-site docking onto an active RTK (den Hertog and Hunter, 1996; Lowenstein et al., 1992). In cells with endogenous GRB2 depleted, expression of the GRB2 SH2 domain alone (lacking SH3 domains) also failed to rescue EML4-ALK granule formation, suggesting an additional role for the GRB2 SH3 domains in promoting condensate assembly. Expression of the double SH3 mutant form of GRB2 in this system resulted in a partial rescue of EML4-ALK granule formation (Figures 4J and 4K), without restoring downstream signaling (Figure 4L) as might be expected due to reduced SOS1 binding at both GRB2 SH3 domains (Cussac et al., 1994; Li et al., 1993; Rozakis-Adcock et al., 1993). These data suggest that GRB2 SH3 domains may promote granule formation by increasing valency through recruitment of additional interacting proteins beyond SOS1 and GAB1, because knockdowns of either gene did not impact granule formation (Figures 4G and 4H), and/or via structural features intrinsic to the SH3 domains that promote higher-order protein assembly. In total, our findings reveal the structural basis of EML4-ALK protein granule assembly that requires contributions from both the fusion partner (via the trimerization domain and truncated TAPE domain in EML4) and the kinase (indirectly through phospho-site docking of the multivalent adaptor protein GRB2 onto phosphorylated EML4-ALK).

### Higher-order assembly of an RTK in membraneless cytoplasmic protein granules is sufficient to activate RAS/MAPK signaling

**Figure S5:**
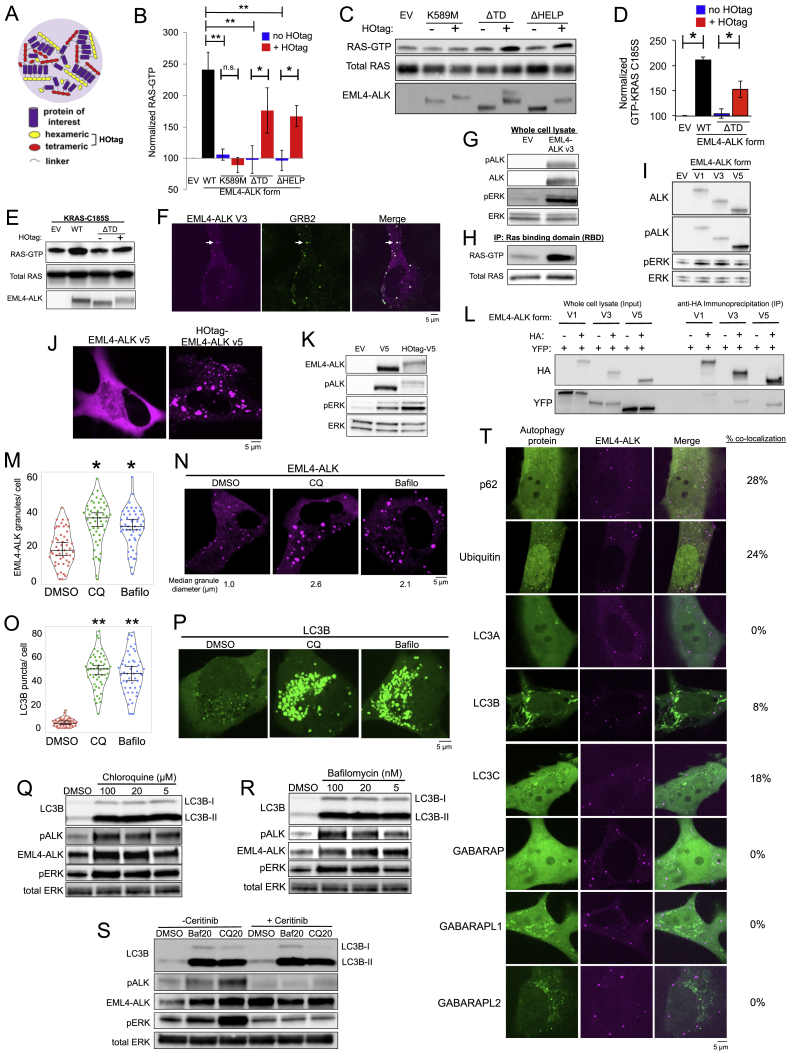
Higher-order granule formation is critical for RAS/MAPK signaling, related to Figure 5 (A) Forced clustering of proteins achieved by N-terminal hexameric and C-terminal tetrameric tags that form higher-order clustered assemblies upon expression in cells. (B, C) Quantification of endogenous RAS-GTP levels and representative western blots from 293T cells expressing an empty vector (EV), EML4-ALK wild-type (WT), or the diffusely cytosolic EML4-ALK mutants (kinase deficient K589M, ΔTD, or ΔHELP) +/– forced clustering (HOtag). EML4-ALK K589M, ΔTD and ΔHELP mutants (blue bars) display significantly reduced RAS-GTP levels compared to wild-type EML4-ALK (black bar), ^∗∗^ denotes p < 0.01 by one-way ANOVA with post hoc Tukey’s HSD test. Forced clustering (HOtag, red bars) of EML4-ALK ΔTD and ΔHELP mutants, but not EML4-ALK K589M, significantly increases RAS-GTP levels compared to the respective non-clustered EML4-ALK mutants (blue bars), ^∗^ denotes p < 0.05, n.s. denotes non-significant comparison, paired t test. RAS-GTP levels normalized to total RAS protein levels and then internally normalized to EML4-ALK mutant expression levels. n = 4. Error bars represent ± SEM. (D, E) Stable expression of cytosolic KRAS mutant (KRAS C185S) in 293T cells, followed by transfection of empty vector (EV), EML4-ALK wild-type (WT) or diffusely cytosolic EML4-ALK ΔTD mutant ± forced clustering (HOtag). Levels of GTP-bound KRAS C185S were normalized to total KRAS C185S protein levels and then internally normalized to expression level of EML4-ALK form. Western blot images representative of n = 4 independent experiments. Error bars represent ± SEM, ^∗^ denotes p < 0.05 by paired t test. (F) Live-cell confocal imaging of mTagBFP2::EML4-ALK variant 3 expressed in human epithelial cell line Beas2B with endogenous mNG2-tagging of GRB2. White arrows indicate a representative EML4-ALK variant 3 cytoplasmic protein granule with local enrichment of GRB2 (multiple non-highlighted granules also show colocalization between EML4-ALK variant 3 and GRB2). Images are representative of at least 25 analyzed cells in 3 independent experiments. (G, H) Western blot analysis upon expression of empty vector (EV) or EML4-ALK variant 3 in 293T cells to assess levels of EML4-ALK activation (phosphorylation), ERK activation, and in immunoprecipitation (IP) panel (H), levels of RAS activation (GTP-bound RAS). Representative images from at least 4 independent experiments. (I) Western blot analysis upon expression of empty vector (EV) or EML4-ALK variants 1, 3, or 5 in 293T cells to assess levels of ERK activation. Images are representative of at least 4 independent experiments and pERK levels are quantified in Main Figure 5J. (J) Live-cell confocal imaging of YFP::EML4-ALK variant 5 ± forced clustering (HOtag) in human epithelial cell line Beas2B. Images are representative of at least 20 analyzed cells in 3 independent experiments. (K) Western blot analysis upon expression of empty vector (EV) or EML4-ALK variant 5 ± HOtag in 293T cells. Images are representative of at least 4 independent experiments. (L) YFP- and HA-tagged versions of EML4-ALK variants 1, 3, and 5 were expressed in 293T cells as indicated. Western blot analysis of input and immunoprecipitation (IP) with anti-HA beads. Images are representative of 3 independent experiments. (M, N) Violin plot of EML4-ALK granule number and representative live-cell confocal images of Beas2B cells expressing YFP::EML4-ALK treated with DMSO, 20 μM chloroquine (CQ), or 20 nM bafilomycin (Bafilo) for 24 hours. At least 50 cells were analyzed per condition over 3 independent experiments. Median diameter of granules calculated from analysis of 20 cells. ^∗^ denotes p < 0.05 by one-way ANOVA with post hoc Tukey’s HSD test. (O, P) Violin plot of number of LC3B puncta and representative live-cell confocal images of Beas2B cells expressing mEGFP::LC3B treated with DMSO, 20 μM chloroquine (CQ), or 20 nM bafilomycin (Bafilo) for 24 hours. At least 50 cells were analyzed per condition over 3 independent experiments. ^∗∗^ denotes p < 0.01 by one-way ANOVA with post hoc Tukey’s HSD test. (Q, R, S) Western blot analysis in EML4-ALK expressing H3122 cancer cell line treated with listed doses of chloroquine or bafilomycin for 24 hours. 500 nM ceritinib was added 1 hour prior to harvesting lysates (S). Images are representative of 4 independent experiments. (T) Live-cell confocal images of Beas2B cells expressing mTagBFP2::EML4-ALK and mEGFP-tagged LC3/ATG8 family proteins. Images are representative of 3 independent experiments. Percent colocalization calculated from at least 60 cells in a total of 3 independent experiments. Standard error for percent colocalization: p62 (2.1), Ubiquitin (4.0), LC3B (0.9), and LC3C (2.6).

Biomolecular condensates, such as EML4-ALK protein granules, are typically micron-sized, membraneless, higher-order protein assemblies (Banani et al., 2017). Using structural mutants of EML4-ALK, we demonstrated that disruption of protein granule formation impaired RAS/MAPK pathway activation. We therefore hypothesized that higher-order assembly of an RTK in membraneless cytoplasmic protein granules is sufficient to organize activation of RAS/MAPK signaling. To directly test this hypothesis, we utilized the HOtag method developed recently to enable forced protein granule formation through multivalent interactions that drive higher-order protein assembly (Zhang et al., 2018) (Figure S5A).

**Figure 5:**
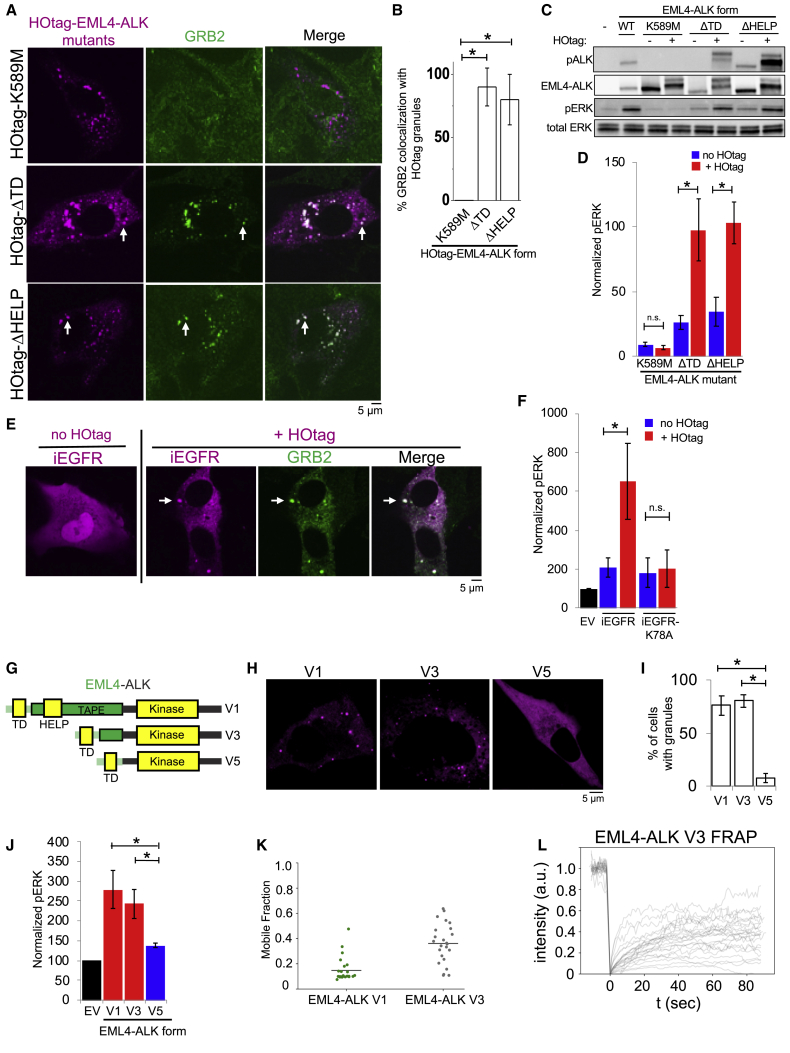
Forced higher-order assembly of EML4-ALK cytosolic mutants drives RAS/MAPK signaling (A) Live-cell imaging of HOtag-mTagBFP2::EML4-ALK mutants in Beas2B cells with endogenous mNG2-tagging of GRB2. (B) Quantification of GRB2 colocalization with HOtag-EML4-ALK mutant granules. 130 total cells per condition, n = 3. (C and D) Western blotting upon expression of WT or mutant EML4-ALK forms ± HOtag in 293T cells, n = 5. pERK levels normalized to WT EML4-ALK, set at 100. (E) Live-cell imaging of mTagBFP2::iEGFR ± HOtag in Beas2B cells with endogenous mNG2-tagging of GRB2. (F) Quantification of western blotting upon expression of EV, iEGFR, or iEGFR kinase-deficient (K78A) mutant ± HOtag in 293T cells, n = 6. (G) Structure schematic of EML4-ALK variants 1, 3, and 5. (H and I) Live-cell imaging of YFP::EML4-ALK variants in Beas2B cells (H). Quantification of % cells with granules (6 or greater) (I). 100+ total cells per condition, n = 3. (J) Quantification of western blotting upon expression of EML4-ALK variants in 293T cells. n = 3. (K) Quantification of FRAP for YFP::EML4-ALK variants in Beas2B cells. Mobile fraction refers to % recovery at 1 min. EML4-ALK variant 1 (N = 25 cells) and variant 3 (N = 24 cells), conducted over 3 replicates. (L) FRAP analysis of YFP::EML4-ALK variant 3 granules in Beas2B cells. N = 24 cells. For (A) and (E), arrows indicate representative HOtag granules with local enrichment of GRB2 (multiple non-highlighted granules also show colocalization). For all panels, error bars represent ±SEM, ^∗^p < 0.05. n.s., non-significant comparison, one-way ANOVA with post hoc Tukey’s HSD test (B, I, and J) or paired t test (D and F). See also Figure S5.

HOtag-induced cytoplasmic granule formation of either the ΔTD or ΔHELP mutants of EML4-ALK locally recruited GRB2 (Figures 5A and 5B), increased RAS-GTP levels (Figures S5B and S5C), and restored RAS/MAPK signaling (Figures 5C and 5D). As an important negative control, HOtag-forced clustering of the kinase-deficient EML4-ALK did not promote GRB2 recruitment or RAS/MAPK signaling (Figures 5A–5D, S5B, and S5C). The findings highlight the dual importance of cytoplasmic protein granule formation and intact kinase activity for productive signaling. We also directly tested the role of protein granule formation on cytosolic RAS activation. Compared to wild-type EML4-ALK, the ΔTD mutant that is distributed diffusely in the cytosol demonstrated substantially reduced levels of activated (GTP-bound) cytosolic KRAS-C185S, which could be restored through HOtag-forced clustering (Figures S5D and S5E). Collectively, our data show that membraneless EML4-ALK cytoplasmic protein granules can spatially concentrate, organize, and initiate RAS/MAPK pathway signaling events.

We tested the generality of this model for membraneless RTK signaling. As a proof-of-principle experiment to test the functional importance of higher-order protein assembly for RTK signaling, we engineered an intracellular EGFR (iEGFR) protein lacking the native extracellular and transmembrane domains. This iEGFR is similar to naturally occurring truncated forms of this RTK and others (Liao and Carpenter, 2012; Ni et al., 2001) and is distributed diffusely in the cytoplasm and nucleus when expressed alone (Figure 5E). HOtag-forced clustering of iEGFR-recruited GRB2 and increased RAS/MAPK signaling in a kinase-dependent manner, analogous to oncogenic ALK (Figures 5E and 5F). These data support a general principle that membraneless higher-order protein assembly of RTKs can organize productive RAS/MAPK signaling.

### Higher-order protein granule formation is functionally distinct from lower-order multimerization and critical for robust signaling output

In order to define the structural rules governing higher-order protein assembly, we examined a set of naturally occurring EML4-ALK variants that have been described in cancer patients (Sabir et al., 2017). All EML4-ALK variants contain the intracellular domain of ALK (but not its transmembrane domain) fused to N-terminal fragments of EML4 of varying lengths (Figure 5G). We observed that another recurrent form of oncogenic EML4-ALK (variant 3), which contains a further truncation of the TAPE domain but retains the TD (Sabir et al., 2017), also formed cytoplasmic granules that locally recruited GRB2 and increased RAS/MAPK signaling (Figures 5H–5J and S5F–S5I). Protein granules formed by EML4-ALK variant 3 demonstrate more liquid-like biophysical properties than those formed by EML4-ALK variant 1 based on FRAP experiments (Figures 5K and 5L). Both variants activated RAS/MAPK signaling with similar potency (Figures 5J and S5I), highlighting the functionality of biomolecular condensates across the continuum of solid-like and liquid-like biophysical states (Alberti et al., 2019; Belyy et al., 2020; Woodruff et al., 2017).

In contrast, EML4-ALK variant 5, which lacks the entire TAPE domain of EML4, did not form visible higher-order protein granules. EML4-ALK variant 5 demonstrated substantially less RAS/MAPK signaling compared to the higher-order protein granule-forming EML4-ALK variants 1 and 3 (Figures 5J and S5I). HOtag-forced clustering of EML4-ALK variant 5 promoted higher-order granule formation and augmented RAS/MAPK signaling (Figures S5J and S5K). Consistent with the presence of TD in all EML4-ALK variants, the granule-forming EML4-ALK variants (1 and 3) and the non-granule-forming variant 5 were each capable of self-association in co-immunoprecipitation experiments (Figure S5L). These results reinforce the role of higher-order protein assembly as a critical feature of robust oncogenic signaling by these RTK fusion oncoproteins. The findings also identify a functional difference between the high-level RAS/MAPK signaling output from protein granule-forming variants (EML4-ALK variants 1 and 3) versus the lower-level signaling output from variants only capable of lower-order self-association through oligomerization (EML4-ALK variant 5).

Last, we reasoned that if higher-order structures are important for robust downstream signaling, then blocking the disassembly and degradation of the RTK protein granules should augment downstream signaling output. Because autophagy is one established mechanism of native RTK regulation and turnover (Bell et al., 2019; Sandilands et al., 2012), we tested whether blocking autophagy impacted RTK protein granule properties and signaling. We found that a subset of EML4-ALK cytoplasmic protein granules were subject to autophagic regulation (Figures S5M–S5T). Autophagic blockade increased both EML4-ALK granule number and size, resulting in significantly increased EML4-ALK activation (transphosphorylation) and RAS/MAPK signaling output (Figure S5M–S5R). ALK inhibitor treatment reversed the increased RAS/MAPK signaling caused by autophagy blockade (Figure S5S), reinforcing the active kinase function of these structures that are regulated by autophagy. These data further demonstrate that higher-order RTK oncoprotein assemblies are potent drivers of downstream RAS/MAPK signaling and identify a role for autophagy in the degradation of higher-order EML4-ALK protein granules that are actively signaling.

### Cytoplasmic granule formation is a general mechanism for oncogenic RTK-mediated RAS/MAPK pathway activation

**Figure 6:**
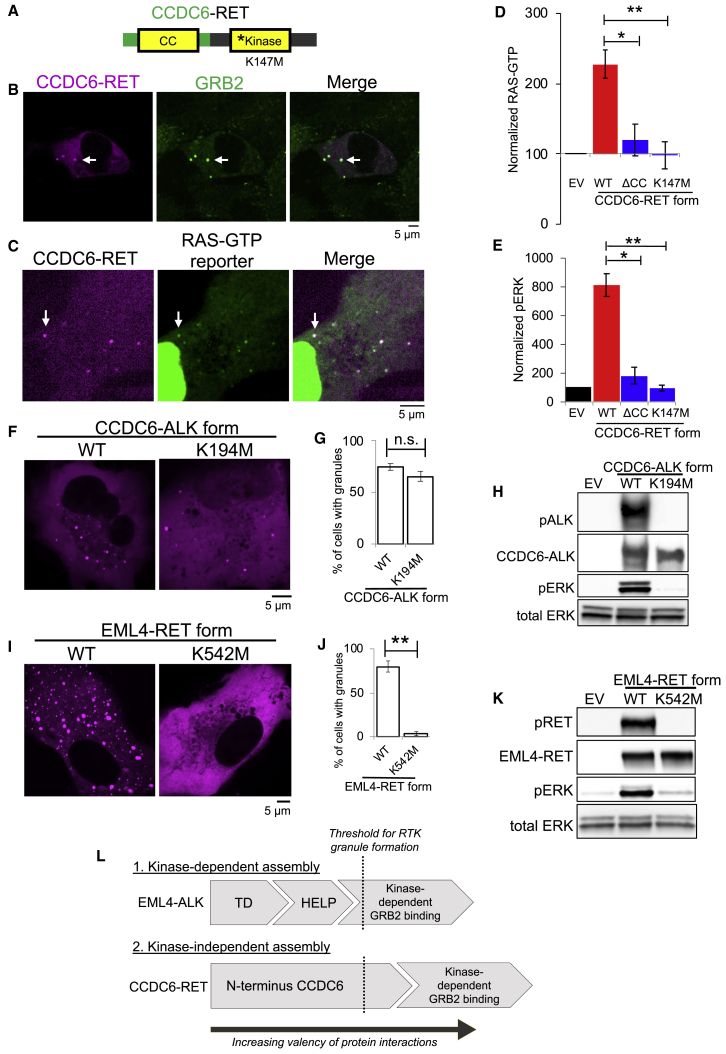
Cytoplasmic granule formation is a general mechanism for RTK-mediated RAS/MAPK pathway activation in cancer (A) Domain structure schematic of CCDC6-RET. (B) Live-cell imaging of mTagBFP2::CCDC6-RET in Beas2B cells with endogenous mNG2-tagging of GRB2. (C) Live-cell imaging of Beas2B cells expressing mTagBFP2::CCDC6-RET and RAS-GTP reporter. (D and E) Quantification of endogenous RAS-GTP levels (D) and ERK phosphorylation (E) by western blotting in 293T cells expressing EV, CCDC6-RET WT, or mutant forms. n = 4. (F and G) Live-cell imaging of mEGFP-tagged CCDC6-ALK WT or kinase-deficient K194M mutant in Beas2B cells. Quantification of % cells with granules (6 or greater) from 75 total cells, n = 3. (H) Western blotting upon expression of EV, CCDC6-ALK WT or K194M mutant in 293T cells. (I and J) Live-cell imaging of mEGFP-tagged EML4-RET WT or kinase-deficient K542M mutant in Beas2B cells. Quantification of % cells with granules from 75 total cells, n = 3. (K) Western blotting upon expression of EV, EML4-RET WT, or K542M mutant in 293T cells. (L) Simplified threshold model for RTK protein granule formation based on cumulative valency of protein interactions contributed by N-terminal RTK fusion partner and kinase-dependent GRB2 binding in each protein assembly context. For (B) and (C), arrows indicate a representative CCDC6-RET cytoplasmic protein granule with local enrichment of GRB2 (B) or RAS-GTP reporter (C) (multiple non-highlighted granules also show colocalization). For all panels, microscopy images representative of at least 75 cells analyzed over 3 replicates. Error bars represent ±SEM, ^∗∗^p < 0.01, ^∗^p < 0.05. n.s., non-significant comparison, one-way ANOVA with post hoc Tukey’s HSD test (D and E) or paired t test (G and J). See also Figure S6.

**Figure S6:**
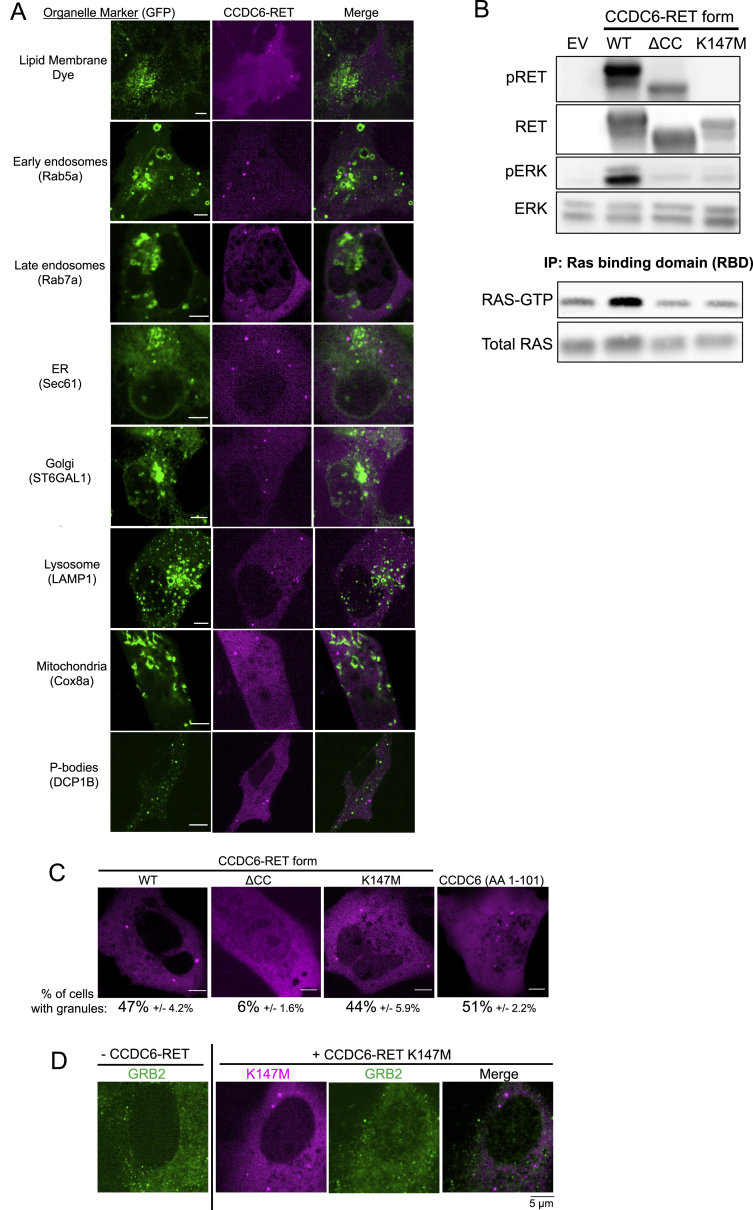
CCDC6-RET forms membraneless cytoplasmic protein granules that are critical for RAS/MAPK pathway activation, related to Figure 6 (A) Live-cell confocal imaging of human epithelial cell line Beas2B upon expression of mTagBFP2::CCDC6-RET and mEGFP-tagged organelle markers as listed. Membrane dye experiments were conducted using live cells incubated with CellTracker CM-DiI Dye (Invitrogen) according to manufacturer’s recommended protocol. Each panel is a representative image of at least 20 analyzed cells per condition with at least 3 independent replicates. (B) Western blotting and immunoprecipitation (IP) for levels of endogenous RAS activation (RAS-GTP) upon expression of an empty vector (EV), CCDC6-RET wild-type (WT) or CCDC6-RET mutants (coiled-coiled domain deletion mutant, ΔCC, and kinase-deficient mutant K147M) in 293T cells. Western blot images are representative of 4 independent experiments. RAS-GTP levels and pERK levels are quantified in Main Figures 6D and 6E. (C) Live-cell confocal imaging of human epithelial cell line Beas2B expressing mTagBFP2-labeled CCDC6-RET wild-type(WT), the coiled-coiled domain deletion mutant (ΔCC), CCDC6-RET K147M (kinase-deficient mutant), or Amino Acids 1-101 of CCDC6. Quantification of cells with 6 or more granules shown as a fraction ± SEM based on 3 independent experiments of at least 25 cells analyzed per condition. Scale bar = 5 μM. (D) Live-cell confocal imaging of mTagBFP2::CCDC6-RET K147M (kinase-deficient mutant) expressed in the human epithelial cell line Beas2B with endogenous mNG2-tagging of GRB2. Images are representative of 60 analyzed cells in total over 3 independent experiments with no observed local enrichment of GRB2 at CCDC6-RET K147M granules.

We further tested the generality of our findings. Similar to ALK, another oncogenic RTK, RET, also undergoes multiple distinct and recurrent gene rearrangements in human cancer, leading to the elimination of the extracellular and transmembrane domains from the various fusion oncoproteins (Kato et al., 2017). The fusion oncoprotein CCDC6-RET formed *de novo* cytoplasmic protein granules that did not demonstrate PM localization or colocalize with intracellular lipid-containing organelles or a lipid-intercalating dye (Figures 6A, 6B, and S6A). CCDC6-RET cytoplasmic protein granules recruited GRB2 (Figure 6B) and locally enriched RAS-GTP as measured by the tandem GFP-RBD reporter (Figure 6C), resulting in increased RAS activation and downstream MAPK signaling (Figures 6D, 6E, and S6B). Structure-function studies showed that a CCDC6-RET mutant lacking the coiled-coil domain in the CCDC6 component (ΔCC) abrogated granule formation but not RET transphosphorylation (Figures S6B and S6C), suggesting that other parts of the N terminus of CCDC6 can mediate lower-order multimerization. Consistent with the functional importance of higher-order protein assembly, the ΔCC mutant reduced RAS/MAPK activation (Figures 6D, 6E, and S6B). A kinase-deficient (K147M) mutant form of CCDC6-RET still formed cytoplasmic protein granules and yet was unable to recruit GRB2 (Figure S6D) or activate RAS/MAPK signaling (Figures 6D, 6E, and S6B). These results reinforce the dual importance of higher-order cytoplasmic protein granules and kinase activity in driving oncogenic RTK/RAS/MAPK signaling.

Our findings also reveal differences between RTK fusion oncoprotein subtypes (i.e., EML4-ALK and CCDC6-RET) in the dependence on kinase activity for granule formation. To investigate why CCDC6-RET protein granule formation is kinase-independent, we swapped the fusion partners EML4 and CCDC6 to generate two additional oncogenic RTK fusion oncoproteins that are also present in human cancers, EML4-RET and CCDC6-ALK (Hillier et al., 2019; Iams and Lovly, 2018). Both EML4-RET and CCDC6-ALK formed cytoplasmic protein granules that activated RAS/MAPK signaling (Figures 6F–6K), providing additional naturally occurring examples of higher-order membraneless protein assembly by RTK fusion oncoproteins. CCDC6-ALK granule formation was kinase-independent (phenocopying CCDC6-RET), whereas EML4-RET granule formation required kinase activity (phenocopying EML4-ALK) (Figures 6F–6K). Moreover, the N terminus of CCDC6 alone, containing the coiled-coil domain, formed protein granules to similar levels as CCDC6-RET itself (Figure S6C). These data demonstrate that structural features present within the N terminus of CCDC6 are sufficient to promote higher-order protein granule formation, explaining why CCDC6-containing RTK fusions can undergo higher-order protein assembly independent of kinase activity. In contrast, the EML4 structural motifs (TD and truncated TAPE domain) are necessary, but not sufficient, for granule formation and thus require additional multivalent contributions arising from kinase-dependent GRB2 phospho-site docking onto EML4-ALK. These structural studies provide an initial set of rules that govern higher-order protein assembly by RTK fusion oncoproteins (Figure 6L).

### Signaling protein architecture and downstream signaling outputs of cytoplasmic RTK protein granules reveal a distinct membraneless subcellular platform for RTK signaling

**Figure 7:**
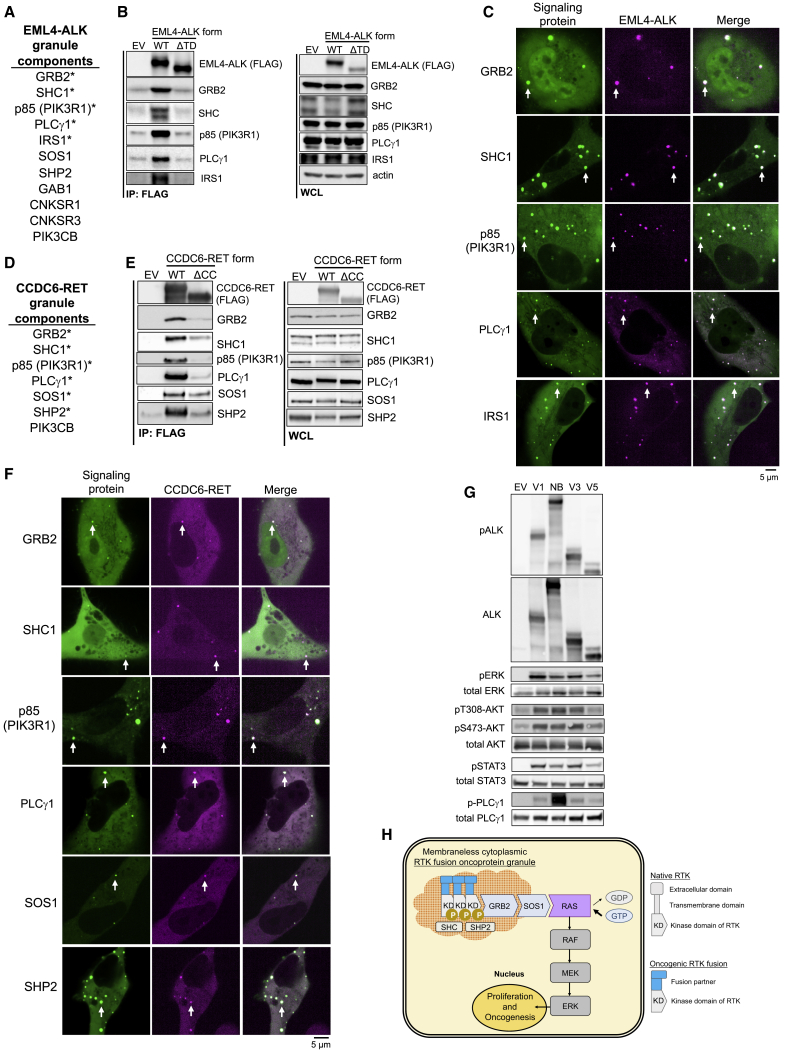
Signaling architecture of membraneless RTK protein granules (A) List of EML4-ALK granule components. (B) Anti-FLAG co-IP of FLAG-tagged EML4-ALK WT or ΔTD mutant expressed in 293T cells, followed by western blotting. (C) Live-cell imaging of mTagBFP2::EML4-ALK and mEGFP-tagged signaling proteins expressed in Beas2B cells. (D) List of CCDC6-RET granule components. (E) Anti-FLAG co-IP of FLAG-tagged CCDC6-RET WT or ΔCC mutant expressed in 293T cells, followed by western blotting. (F) Live-cell imaging of mTagBFP2::CCDC6-RET and mEGFP-tagged signaling proteins expressed in Beas2B cells. (G) Western blotting upon expression in 293T cells of EML4-ALK variants or oncogenic full-length ALK (F1174L) found in neuroblastoma (NB). n = 4. (H) Model for membraneless cytoplasmic protein granule-based oncogenic RTK/RAS/MAPK signaling. KD denotes the kinase domain of the RTK fusion oncoprotein. For (A), (B), (D), and (E), starred proteins both co-precipitate and locally enrich by imaging, non-starred proteins only enrich at granules by imaging. WCL denotes whole cell lysates. Images representative of at least 4 replicates. For (C) and (F), arrows indicate a representative EML4-ALK or CCDC6-RET protein granule with local enrichment of respective signaling proteins (multiple non-highlighted granules also show colocalization). All listed signaling proteins showed greater than 85% colocalization with EML4-ALK or CCDC6-RET granules. n = 3. See also Figure S7.

**Figure S7:**
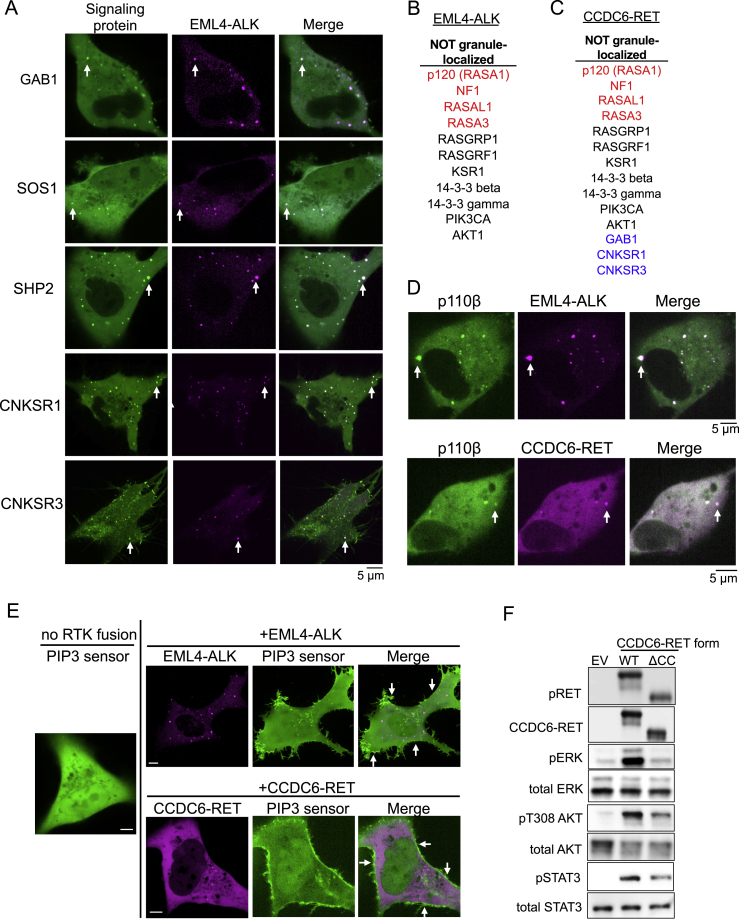
Signaling components of membraneless RTK protein granules, related to Figure 7 (A) Live-cell confocal imaging of mTagBFP2::EML4-ALK and mEGFP-tagged signaling proteins expressed in Beas2B cells. White arrows indicate a representative EML4-ALK protein granule with local enrichment of respective signaling proteins (multiple non-highlighted granules also show colocalization between EML4-ALK and signaling proteins). Images are representative of at least 20 cells analyzed in 3 independent experiments with greater than 80% colocalization observed for all signaling proteins. (B, C) List of proteins that were mEGFP-tagged and did not enrich at mTagBFP2::EML4-ALK or mTagBFP2::CCDC6-RET protein granules upon expression in Beas2B cells. RAS GTPase activating proteins (GAPs) are displayed in red, proteins with differential localization between EML4-ALK and CCDC6-RET are displayed in blue. (D) Live-cell confocal imaging of mEGFP::p110β and mTagBFP2::EML4-ALK or mTagBFP2::CCDC6-RET expressed in Beas2B cells. White arrows indicate a representative RTK protein granule with local enrichment of p110β (multiple non-highlighted granules also show enrichment of p110β). (E) Live-cell confocal imaging of PI3K activity reporter (mCherry-tagged AKT2-PH domain, which functions as a PIP3 sensor) alone or with mTagBFP2::EML4-ALK or mTagBFP2::CCDC6-RET expression in Beas2B cells. White arrows indicate plasma membrane enrichment of the PI3K activity reporter. (F) Western blotting upon expression of wild-type CCDC6-RET or non-granule-forming, coiled-coil domain deletion mutant ΔCC in 293T cells. EV denotes empty vector. Images are representative of at least 5 independent experiments.

We next sought to define and classify the broader signaling protein architecture of membraneless RTK granules. We performed candidate-based co-immunoprecipitation experiments and cellular imaging-based screening for granule enrichment of canonical signaling proteins involved in RTK and RAS signaling. Consensus RTK granule components included GRB2, SHC1, PIK3R1 (p85), and PLCγ1, which co-precipitated with both EML4-ALK and CCDC6-RET in a granule-dependent manner and localized to RTK cytoplasmic granules (Figures 7A–7F). We also identified SHP2, SOS1, GAB1, and the scaffold proteins IRS1, CNKSR1, and CNKSR3 as granule-enriched components, although with more variability across methods and between RTK fusion oncoproteins (Figures 7A–7F and S7A). Finally, we found that a number of notable signaling proteins in the RAS/MAPK pathway do not enrich at RTK protein granules, including several negative regulators of RAS activation such as the RAS GTPase activating proteins (GAPs) p120, NF1, RASAL1, and RASA3 (Figures S7B and S7C). The clear presence of RAS-activating proteins and absence of several RAS GAPs provides one potential explanation for the robust RAS/MAPK output emanating from the cytoplasmic RTK protein granules.

Given the signaling protein architecture of membraneless RTK protein granules, we next investigated whether these structures activate other canonical signaling pathways that are downstream of lipid membrane resident RTKs. The selected pathways included phosphoinositide 3-kinase (PI3K)-AKT, Janus kinase (JAK)-signal transducer and activator of transcription (STAT), and phospholipase C (PLC-γ). We directly compared the signaling output from granule-forming EML4-ALK variants (1 and 3) to non-granule-forming EML4-ALK variant 5 and a membrane-resident oncogenic form of full-length ALK. We found that EML4-ALK protein granules activate both cytosolic JAK/STAT and lipid-dependent PI3K/AKT signaling in a granule-dependent manner (Figure 7G). Both the PI3K regulatory subunit, p85, and a catalytic subunit, p110β, were enriched at EML4-ALK protein granules (Figures 7C and S7D). However, cellular PI3K activity, as assayed by an established fluorescent reporter for phosphatidylinositol (3,4,5)-trisphosphate (PIP3) (Ebner et al., 2017), was not increased at membraneless cytoplasmic EML4-ALK protein granules and, instead, was enriched at the PM (Figure S7E). Further, EML4-ALK protein granules displayed minimal activation of lipid-dependent PLC-γ1 compared to the membrane-resident form of oncogenic ALK, despite local recruitment of PLC-γ1 (Figure 7G). CCDC6-RET displayed a similar pattern of granule-dependent activation of JAK/STAT and PI3K/AKT signaling (Figure S7F) and increased PI3K activity at the PM, and not at CCDC6-RET membraneless protein granules (Figure S7E). These data establish the broader signaling protein architecture and outputs of membraneless cytoplasmic RTK protein granules. The findings highlight distinct patterns of downstream signaling pathway activation from these membraneless RTK protein granules and the potential for spatial coordination and crosstalk between membraneless cytoplasmic protein granule and traditional lipid membrane-based RTK signaling platforms.

## Discussion

Collectively, our findings reveal a new mechanism for RTK activation and RAS signaling in cancer. We demonstrate that certain RTK fusion oncoproteins assemble *de novo* their own subcellular compartment, membraneless cytoplasmic protein granules, which coordinate local RAS activation in a lipid membrane-independent manner to drive oncogenic signaling (Figure 7H).

Physiologic RTKs, as well as oncogenic RTKs with kinase-activating missense or small insertion/deletion mutations, are integral membrane proteins that localize to and organize signaling events at lipid membrane subcellular compartments, including the PM and certain intracellular organelles (Lemmon and Schlessinger, 2010). In contrast, RTKs that undergo chromosomal rearrangements in cancer often lose their lipid membrane-targeting sequences (i.e., transmembrane domains) from the RTK fusion oncoprotein (Du and Lovly, 2018). The non-kinase fusion partner frequently contains multimerization domains that are important for self-association and oncogenic signaling (e.g., TD within EML4 in EML4-ALK) (Du and Lovly, 2018; McWhirter et al., 1993; Soda et al., 2007). However, it was not known whether RTK fusion oncoproteins form higher-order protein assemblies or whether multimerization alone was sufficient to drive oncogenic signaling. Here, we report several initial examples of RTK fusion oncoproteins forming biomolecular condensates that are critical for oncogenic RTK/RAS signaling. We determined that the presence of a multimerization domain alone is not sufficient for protein granule formation (non-granule-forming EML4-ALK variant 5 contains a TD) and identify a significant functional difference (increased signaling output) between forms of EML4-ALK and CCDC6-RET that are capable of higher-order protein assembly compared to those that are only competent for lower-order self-association.

We propose that *de novo* assembly of membraneless cytoplasmic protein granules may be a general mechanism for activating RTK fusion oncoprotein signaling in cancer. This mechanism is distinct from other known strategies of RTK activation including promoter driven overexpression of the oncoprotein or dimerization/oligomerization mediated by domains within the non-kinase fusion partner (Du and Lovly, 2018; Medves and Demoulin, 2012). Here, we describe an initial set of principles that govern whether individual RTK fusion oncoproteins can form membraneless cytoplasmic protein granules. First, the structural and biophysical features contributed by the non-kinase fusion partner (e.g., EML4 or CCDC6) are important determinants of granule formation. The N terminus of CCDC6 and the forced granule system (HOtag) were sufficient to drive higher-order protein assembly, in contrast to the N terminus of EML4 where the TD and HELP domains were necessary, but not sufficient, for granule formation. Second, kinase-dependent phospho-site docking of adaptor proteins such as GRB2 can contribute additional multivalent interactions to promote condensate formation, in cases where the structural features of the non-kinase fusion partner are insufficient (e.g., EML4). Our results suggest that one organizational framework for RTK fusion oncoprotein assemblies is whether condensate formation is kinase-dependent (Figure 6L). Importantly, our findings demonstrate that robust downstream signaling emanating from RTK oncoprotein granules requires more than kinase function and lower-order oligomerization; our findings here establish the critical role of higher-order protein assembly. Additional structural rules governing RTK granule formation such as the effects of individual domains on intrinsic solubility, the number and types of multivalent interaction domains required for higher-order assembly, and the determinants of granule size remain to be fully defined.

Downstream of RTKs, RAS activation is a pathogenic hallmark of many cancers driven by chimeric RTK oncoproteins (Rotow and Bivona, 2017). The current paradigm holds that RAS proteins (e.g., H/N/K-RAS) are activated and signal to effector proteins such as the RAF/MEK/ERK kinases exclusively from lipid membrane compartments in mammalian cells (Cox et al., 2015; Willumsen et al., 1984). How naturally occurring chimeric RTK oncoproteins that lack lipid membrane-targeting domains spatially coordinate RAS activation remained unclear. Our findings provide first examples of RAS activation and productive downstream signaling from a membraneless subcellular compartment in mammalian cells. RAS proteins undergo dynamic exchange between the cytoplasm and lipid membrane subcellular compartments including the plasma membrane, endosomes, and Golgi apparatus (Cox et al., 2015). Our data suggest a functional role for the cytoplasmic pool of RAS, although we do not exclude signaling contributions from lipid membrane-based pools of RAS. Moreover, isoform-specific differences in RAS activation by membraneless RTK protein granules (i.e., increased cytosolic KRAS activation compared to HRAS and NRAS) suggest isoform-specific features, like the polylysine track of KRAS4B, may regulate dynamic exchange between the cytosol, lipid membrane compartments, and membraneless RTK granules. These results offer an alternative solution by which cells can organize oncogenic RTK/RAS/MAPK signaling that is distinct from canonical lipid membrane platforms such as the PM.

RTKs can utilize an array of adaptor and effector proteins to regulate outputs from multiple signaling pathways (Lemmon and Schlessinger, 2010). In addition to the RAS/MAPK pathway, we demonstrate RTK protein granule-dependent activation of cytoplasmic JAK/STAT and lipid-based PI3K/AKT signaling, but not lipid-based PLC-γ signaling. The activation of PI3K/AKT signaling by membraneless RTK protein granules is surprising given that PI3K requires a phospholipid substrate. Our data indicate that PI3K activation and substrate engagement may occur at distinct subcellular compartments. We observe that the regulatory and catalytic subunits of PI3K are locally enriched at membraneless RTK protein granules. However, PI3K activity itself is enriched not at the RTK protein granule, but instead at the PM. How signaling emanating from membraneless RTK granule-based activation of lipid effectors (or lack thereof in the case of PLC-γ) is transmitted to canonical lipid membrane platforms prompts important questions regarding signal transduction compartmentalization and crosstalk between biomolecular condensates and lipid membrane platforms. Although our data suggest that the signaling protein architecture and output of RTK membraneless cytoplasmic protein granules may differ in important ways from lipid membrane-based RTK signaling complexes, the functional significance of protein granule-based and membrane-based signaling crosstalk remains to be determined.

We provide an initial architectural view of the signaling proteins and regulation operative at membraneless cytoplasmic protein granules. Our study identifies multiple RTK adaptor proteins including SHC1, IRS1, and GAB1 at RTK protein granules, as well as the RAS activating proteins SHP2 and SOS1. How do RTK protein granules preferentially engage with or accelerate specific signaling effector cascades such as the RAS/MAPK pathway? One hypothesis for this property is by increasing the local concentration of the RTK itself as well as RAS adaptor proteins, cytoplasmic protein granules may shift the balance of RAS GTP/GDP exchange toward RAS-GTP (i.e., activation) and MAPK signaling in cancer cells. Consistent with this notion, we observed enrichment of RAS activating proteins (e.g., SOS1 and others) at RTK protein granules and yet no substantial enrichment of RAS GAPs, which are negative regulators of RAS signaling. Alternatively, cytoplasmic protein granules may sequester specific signaling proteins and alter the equilibrium between cytoplasmic granule and lipid membrane-based signaling protein pools (e.g., the enrichment of GRB2 at the granules). How the biophysical state of RTK granules (more solid-like or liquid-like) impacts RAS/MAPK signaling output also remains to be determined. Our data support a consensus model of a continuum between biophysical states (Alberti et al., 2019) and demonstrates functionality in terms of signaling output from both more solid-like and more liquid-like RTK granules (EML4-ALK variants 1 and 3).

In summary, we report on the discovery of a cancer-specific membraneless subcellular structure formed by higher-order assembly of an RTK oncoprotein that is critical for oncogenic RTK/RAS signaling. Our results provide rationale for a new class of targeted therapeutics that aim to disrupt protein granule assembly and function. Treatment for oncogenic RTK/RAS/MAPK-driven cancers is almost universally characterized by the development of drug resistance to targeted kinase inhibitors (Rotow and Bivona, 2017). Identifying critical factors that regulate the nucleation and degradation of RTK membraneless cytoplasmic protein granules, as well as defining roles for molecular chaperones and signaling proteins that promote multivalency-driven condensate formation, may provide opportunities for the development of a distinct class of targeted drugs to disrupt protein granules that drive cancer pathogenesis.

### Limitations of study

The limitations of this study relate to the broader functional relevance of membraneless RTK and RAS signaling. The absence of a membrane targeting domain is a shared structural feature of many RTK fusion oncoproteins (Nelson et al., 2017), and we demonstrate that multiple prominent examples of RTK fusion oncoproteins form membraneless cytoplasmic protein granules. However, the prevalence of membraneless higher-order protein assembly within the broader class of RTK fusion oncoproteins remains to be determined. There are also mechanistic questions relating to RAS activation and downstream RAF/MEK/ERK signaling in the absence of a lipid membrane that remain to be explored. For example, we show that membraneless RTK protein granules locally activate cytoplasmic RAS, and these structures are critical for oncogenic RAS/MAPK signaling output; however, we do not exclude the possibility that cytoplasmic RTK protein granules also activate lipid membrane-based RAS/MAPK signaling, either through the diffusion of activated RAS between cytoplasmic RTK granules and lipid membranes or via direct contact between cytoplasmic RTK protein granules and lipid membranes (PM or internal). Future microscopy-based analysis of RAS and MAPK pathway spatiotemporal dynamics at RTK oncoprotein granules and biochemical reconstitution of RTK protein granules will help further clarify the mechanistic basis of lipid membrane-independent oncogenic RTK/RAS/MAPK signaling. Such studies could reveal important principles of crosstalk between lipid membrane-based and membraneless subcellular compartments. Finally, the possibility that membraneless higher-order assemblies have physiologic roles in native RTK and RAS signaling has not been addressed. We demonstrate productive RAS/MAPK signaling output through forced higher-order assembly of a truncated form of EGFR, similar to naturally occurring forms of this RTK (Liao and Carpenter, 2012), as a proof-of-principle for functional membraneless RTK and RAS signaling. Whether RTK or RAS activating membraneless cytoplasmic protein granules exist in non-transformed cells or are aberrant structures limited to pathogenic processes driven by RTK/RAS/MAPK pathway hyperactivation remains an important area for future investigation.

## STAR★Methods

### Key resources table

**Table:** 

REAGENT or RESOURCE	SOURCE	IDENTIFIER
**Antibodies**		

ALK (D5F3)	Cell Signaling Technology	RRID:AB_11127207
p-Y1604-ALK	Cell Signaling Technology	RRID:AB_331047
ERK1/2	Cell Signaling Technology	RRID:AB_10695739
p-T202/Y204-ERK1/2	Cell Signaling Technology	RRID:AB_2315112
DCP1B (D2P9W)	Cell Signaling Technology	RRID:AB_2798157
EEA1 (C45B10)	Cell Signaling Technology	RRID:AB_2096811
EGF Receptor (D38B1)	Cell Signaling Technology	RRID:AB_2246311
p-Y1068-EGF Receptor (D7A5)	Cell Signaling Technology	RRID:AB_2096270
MEK1/2	Cell Signaling Technology	RRID:AB_823567
p-S221-MEK1/2 (166F8)	Cell Signaling Technology	RRID:AB_490903
RET (C31B4)	Cell Signaling Technology	RRID:AB_2238465
p-Y905-RET	Cell Signaling Technology	RRID:AB_2179887
GFP/YFP (D5.1)	Cell Signaling Technology	RRID:AB_1196615
HA (C29F4)	Cell Signaling Technology	RRID:AB_1549585
SHC1	Cell Signaling Technology	RRID:AB_2254631
PLCγ1	Cell Signaling Technology	RRID:AB_10691383
SOS1	Cell Signaling Technology	RRID:AB_10626628
PIK3R1-p85	Cell Signaling Technology	RRID:AB_659889
GAB1	Cell Signaling Technology	RRID:AB_2304999
IRS1	Cell Signaling Technology	RRID:AB_330333
SHP2	Cell Signaling Technology	RRID:AB_2174959
LC3B	Cell Signaling Technology	RRID:AB_915950
HRP-conjugated anti-mouse	Cell Signaling Technology	RRID:AB_330924
HRP-conjugated anti-rabbit	Cell Signaling Technology	RRID:AB_2099233
Actin	Santa Cruz Biotechnologies	RRID:AB_630836
HRAS	Santa Cruz Biotechnologies	RRID:AB_631670
NRAS	Santa Cruz Biotechnologies	RRID:AB_628041
KRAS	Santa Cruz Biotechnologies	RRID:AB_627865
Calnexin	Santa Cruz Biotechnologies	RRID:AB_626783
GRB2	Santa Cruz Biotechnologies	RRID:AB_627693
Anti-Ras, clone 10	EMD-Millipore	RRID:AB_2121151
Anti-FLAG M2 monoclonal	Sigma-Aldrich	RRID:AB_259529
Alexa Fluor 488/594	Abcam	RRID:AB_2576208, RRID:AB_2650602

**Chemicals, peptides, and recombinant proteins**		

Crizotinib	Selleck Chemicals	Cat #S1068
Erlotinib	Selleck Chemicals	Cat #S7786
Triton X-100	Sigma-Aldrich	Cat #T8787
RNase-A	Thermo Fisher Scientific	Cat #12091021
HALT Protease inhibitor cocktail	Thermo Fisher Scientific	Cat #87785
HALT Phosphatase inhibitor cocktail	Thermo Fisher Scientific	Cat #78420
DMSO	Sigma-Aldrich	Cat #D2650
1,6-Hexanediol	Sigma-Aldrich	Cat #240117
Ceritinib	Selleck Chemicals	Cat #S7083
Bafilomycin	Sigma-Aldrich	Cat #B1793
Chloroquine	Sigma-Aldrich	Cat #C6628

**Critical commercial assays**		

RAS GST-RBD Activation Kit	Cytoskeleton	Cat #BK008
MycoAlert Mycoplasma Detection Kit	Lonza	Cat #LT07-118
Amersham ECL Prime Western Blotting Detection Reagent	GE Life Sciences	Cat #RPN2232
Mirus Bio TransIT-LT1	Fisher Scientific	Cat #MIR 2304
M2 agarose flag beads	Sigma Aldrich	Cat #A2220
CellTracker CM-DiI Membrane Dye	Thermo Fisher Scientific	Cat #C7001
Anti-HA beads	Thermo Fisher Scientific	Cat #88836

**Experimental models: Cell lines**		

Beas2B	ATCC	CRL-9609
H3122	Generous gift from Christine Lovly	N/A
STE-1	Generous gift from Christine Lovly	N/A
HCC827	ATCC	Cat #CRL-2868
HEK293T cells	ATCC	Cat #CRL-11268

**Oligonucleotides: sgRNA sequences**		

GRB2	N/A	CTTAGACGTTCCGGTTCACG
SOS1	N/A	ACAGAGGAACTCAGGAAGAA
GAB1	N/A	GCGAAACCGTCCATCTTGCG
KRAS	N/A	AATGACTGAATATAAACTTG
HRAS	N/A	GATGACGGAATATAAGCTGG
NRAS	N/A	AATGACTGAGTACAAACTGG

**Software and algorithms**		

Graphpad Prism 6	Graphpad software	https://www.graphpad.com
ImageJ	Schneider et al., 2012	https://imagej.nih.gov/ij
Micro-Manager software	Edelstein et al., 2010	https://micro-manager.org
CellProfiler software	CellProfiler	https://cellprofiler.org
MATLAB software	Mathworks	https://www.mathworks.com/products/matlab.html

### Resource availability

#### Lead contact

Further information and requests for resources should be directed to and will be fulfilled by the Lead Contact, Trever Bivona (trever.bivona@ucsf.edu).

#### Materials availability

Plasmids generated in this study are available by request from the Lead Contact.

#### Data and code availability

Data and codes generated in this study are available by request from the Lead Contact.

### Experimental model and subject details

#### Cell lines

This study utilized Beas2B, H3122, STE-1, HCC827, and 293T cells. All cell lines were maintained in humidified incubators with 5% CO2 at 37°C. Beas2B and endogenously tagged derivatives, as well as the patient derived cancer cell lines H3122, STE-1, HCC827 were cultured in RPMI-1640 medium supplemented with 10% FBS and penicillin-streptomycin at 100 μg/mL. 293T cells were cultured in DMEM-High Glucose supplemented with 10% FBS and 100 μg/mL of penicillin/streptomycin. All cell lines were tested for mycoplasma every 3 months using MycoAlert Mycoplasma Detection Kit (Lonza, Basel, Switzerland). All cells used were < 20 passages from thaw.

### Method details

#### Cell line generation

Generation of endogenously tagged mNeonGreen2_1-10/11_ cell lines was performed in the human bronchial epithelial cell line (Beas2B) and the patient-derived oncogenic ALK cell line (H3122) as previously described (Feng et al., 2017). Correct integration of mNeonGreen2_11_ was confirmed by genomic sequencing and by reduction in fluorescence upon gene knockdown. sgRNA spacer sequences used in this study are listed in the Key Resources Table.

#### Biochemical fractionation

Cells were seeded in 10 cm dishes and harvested the following day by scraping into buffer A [10 mM Tris-HCl (pH 7.4), 1 mM EDTA, 250 mM Sucrose, supplemented with 1X HALT protease inhibitors (Thermo Fisher Scientific)]. Lysates were gently sonicated on minimum intensity and cleared by centrifugation. Lysate was then split equivalently to two ultracentrifugation tubes, and one tube was supplemented with 1% Triton X-100. Lysates were then subjected to ultracentrifugation at 100,000 × g for 1 hour at 4°C in an Optima MAX Ultracentrifuge (Beckman Coulter, Brea, CA). Supernatant and pelleted fractions were separated, resuspended with Laemmli sample buffer, boiled, and analyzed by SDS-PAGE. For RNase A experiments, lysates were incubated ± RNase A at 1 μg/μL for 30 minutes at room temperature and then subjected to ultracentrifugation and processing as above.

#### Antibodies and immunoblotting

Antibodies against the following were obtained from Cell Signaling Technology (Danvers, MA, USA) and were used at a dilution of 1:1000: ALK (D5F3) (#3633), p-Y1604-ALK (#3341), ERK1/2 (#9107), p-T202/Y204-ERK1/2 D13.14.4e (#4370), DCP1B (#13233), EEA1 (#3288), EGF Receptor (#4267), p-Y1068-EGF Receptor (#3777), MEK1/2 (#9122), p-S221-MEK1/2 (#166F8), RET (#3223), p-Y905-RET (#3221), GFP/YFP (D5.1) (#2956), HA (#3724), SHC1 (#2432), PLCγ1 (#5690), SOS1 (#5890), PIK3R1-p85 (#4257), GAB1 (#3232), IRS1 (#2382),

SHP2 (#3397), LC3B (#2775), horseradish peroxidase (HRP)-conjugated anti-mouse (#7076) and HRP-conjugated anti-rabbit (#7074). Antibodies to the following were obtained from Santa Cruz Biotechnologies (Santa Cruz, CA, USA): actin (I19, 1:1000 dilution), HRAS (C-20, 1:200 dilution), NRAS (F155, 1:200 dilution), KRAS (F234, 1:500 dilution), Calnexin Antibody (AF18), GRB2 (C7: 1:1000). Anti-Ras antibody, clone 10 (1:1000) was obtained from EMD Millipore (Hayward, CA) and anti-FLAG M2 monoclonal antibody was obtained from Sigma (USA).

For immunoblotting, cells were serum-starved (0% serum for 24 hours), then washed with ice-cold PBS and scraped in ice cold RIPA buffer [25 mM Tris⋅HCl (pH 7.6), 150 mM NaCl, 1% NP-40, 1% sodium deoxycholate, 0.1% SDS, supplemented with 1X HALT protease inhibitor cocktail and 1X HALT phosphatase inhibitor cocktail (Thermo Fisher Scientific)]. Lysates were clarified with sonication and centrifugation. Lysates were subject to SDS/PAGE followed by blotting with the indicated antibodies. Signal was detected using Amersham ECL Prime reagent (GE Healthcare Life Sciences, Chicago, IL, USA) and chemiluminescence on an ImageQuant LAS 4000 (GE Healthcare Life Science, Chicago, IL, USA). pERK levels were normalized to total ERK protein levels, displayed relative to EV or control samples, and then internally normalized for the level of expression of RTK fusion oncoprotein mutant/variant forms.

#### Generation of stable cell lines expressing wild-type or cytosolic RAS

293T cells were infected with wild-type H/N/KRAS or respective cytosolic RAS mutants (KRAS C185S, HRAS C186S, NRAS C186S) then selected with puromycin to generate stable cell lines. RAS activation assays were performed 48 hours after transfection of empty vector, EML4-ALK, or oncogenic EGFR (EGFR L858R). H3122 and HCC827 cell lines were also infected with wild-type and C185S KRAS and selected with puromycin to generate stable cell lines. RAS activation assays were performed comparing 2 hour treatment with 100 nM crizotinib in H3122 cells or 100 nM erlotinib in HCC827 cells with mock DMSO treatment.

#### Compounds

Crizotinib, erlotinib, and ceritinib were purchased from Selleck Chemicals (Houston, TX) and resuspended in DMSO.

#### RAS activation assays

The RAS GST-RBD activation kit was obtained from Cytoskeleton (Denver, CO, USA; #BK008). The protocol was according to the manufacturer’s instructions. Lysis buffer for RAS-GTP pulldowns was 50 mM Tris (pH 7.5), 10 mM MgCl_2_, 0.5 M NaCl, and 2% Igepal. 150 μg of lysate was incubated with 10 μL RBD-beads overnight, followed by western blotting. RAS-GTP levels were normalized to relevant total RAS protein levels, standardized against an EV or vehicle control, then internally normalized for the varying expression level of mutant/variant RTK fusion oncoprotein forms.

#### Live-cell microscopy

Cells were seeded in 35mm glass-bottom dishes (MatTek, Ashland, MA) or 8-well Nunc Lab-Tek 8 imaging chambers (Thermo Fisher Scientific) and then imaged using a Nikon Ti microscope with a CSU-W1 spinning disk confocal using a 100X/1.4 Plan Apo VC objective (Nikon Imaging Center, UCSF). Images were acquired on MicroManager software and analyzed using ImageJ software (Edelstein et al., 2014; Schneider et al., 2012).

#### Structured illumination microscopy

Beas2B cells were seeded into Nunc Lab-Tek 8-well imaging chambers and eYFP::EML4-ALK was transfected via Mirus TransIT-LT1 (Mirus Bio LLC, Madison, WI, USA). 24 hours later, both live cells and fixed cells were imaged with structured illumination microscopy on a DeltaVision OMX imaging system (GE Healthcare) in 3D-SIM mode. The procedure for cell fixation was 4% paraformaldehyde incubation for 5 minutes followed by three PBS washes. The 3D structures of granules were rendered with visualization software Chimera X.

#### Fluorescence recovery after photobleaching

For photobleaching experiments, Beas2B cells were seeded into Nunc Lab-Tek 8-well imaging chambers and transfected with eYFP::EML4-ALK variant 1 or 3 via Mirus TransIT-LT1. A 473 nm laser (Rapp Optoelectronic) at excitation intensity of 30 mW was used to photobleach regions of interest (ROIs) corresponding to individual granules in the sample. The fluorescence intensity was monitored before and after photobleaching with time interval of 3 s. Further intensity analyses were done in MATLAB with custom-written code and can be found here: https://github.com/BoHuangLab/EML4-ALK_FRAP.

#### Monitoring granules during hexanediol treatment

Beas2B cells expressing mTagBFP2::EML4-ALK and GFP::DCP1B were seeded into Nunc Lab-Tek 8-well imaging chambers. A custom-made sample holder ensured the imaging chamber fits securely on the microscope stage without position shift. The cells were first imaged in regular RPMI cell culture media and then the media was replaced by RPMI media containing 5% hexanediol (Sigma). The same field of view was monitored at defined time points after addition of hexanediol.

#### Immunoprecipitation

For immunoprecipitation assays, HEK293T cells were transfected with FLAG-tagged versions of EML4-ALK, CCDC6-RET, or respective mutants, or HA- and YFP-tagged versions of EML4-ALK variants 1, 3, and 5. 48 hours post-transfection (after serum starvation in 0% serum for 24 hours), the cells were resuspended in lysis buffer (0.5% NP-40, 150 mM NaCl, 50 mM TrisCl, pH 7.5) containing protease and phosphatase inhibitor cocktails (Sigma). Lysates were syringed and centrifuged to clarify, then whole-cell extracts were either incubated overnight at 4°C with M2 agarose-FLAG beads (Sigma) or for 1 hour with anti-HA beads (Thermo Fischer Scientific). The immunocomplexes were washed three times with wash buffer (50 mM Tris (pH 7.4, 150 mM NaCl) and FLAG or HA beads were boiled and loaded for SDS-PAGE.

#### Immunofluorescence

H3122 or Beas2B cells expressing EML4-ALK were seeded in 4-well Lab Tek II Chamber Slides (Thermo Fisher Scientific). The following day, cells were fixed for 15 minutes with 4% paraformaldehyde, washed, and incubated in blocking buffer for 1 hour (1X PBS with 1% BSA and 0.3% Triton X-100). Blocking buffer was aspirated and cells were incubated with primary antibody (either ALK DF53 1:1000 from Cell Signaling Technology or FLAG-M2 1:1000 from Sigma) overnight in the dark at 4°C. The following day, cells were washed, incubated with fluorophore-conjugated secondary antibodies (Alexa Fluor 488/594 from Abcam, 1:2000) for 1 hour at room temperature in the dark, washed, and then mounted using ProLong Gold Antifade reagent with DAPI (Cell Signaling Technology). Slides were analyzed using a Nikon Ti microscope with a CSU-W1 spinning disk confocal using a 100 × 1.4 NA Plan Apo VC objective (Nikon Imaging Center, UCSF). Images were acquired on MicroManager software and analyzed using ImageJ software.

#### siRNA knockdown and GRB2 mutant rescue experiments

ON Target siRNA smart pools for GRB2, GAB1, SOS1, and non-targeting control were purchased from Dharmacon and used according to manufacturer’s instructions. GRB2-GFP mutants were constructed using QuikChange site-directed mutagenesis (Agilent) using wild-type GRB2-GFP backbone (Addgene plasmid #86873).

#### Autophagy assays

Bafilomycin (DMSO) and chloroquine (water) were purchased from Sigma and resuspended as recommended. GFP-tagged LC3/GABARAP proteins were purchased from Addgene (Plasmid #’s: 123106, 123107, 123110, 123111, 123112, 123113). GFP-tagged p62 and ubiquitin kindly provided by the Center for Advanced Light Microscopy (UCSF).

#### Imaging-based screening of signaling proteins for RTK granule localization

GFP tagging of RTK and RAS pathway genes was performed by Gateway cloning individual genes from the RAS Pathway 2.0 Clone Collection- #1000000070 (Addgene) into an N-terminal GFP Gateway vector. cDNA’s not available through this collection were synthesized by Genewiz. Relevant GFP-tagged signaling proteins were co-expressed with mTagBFP2-tagged EML4-ALK or CCDC6-RET in Beas2B cells and at least 20 cells were manually scored for colocalization in 3 independent experiments.

#### Plasmids and construct generation

EML4-ALK cDNA and respective mutants were cloned into pBabe-puro with a N-terminal FLAG tag and mTagBFP2-C1. HA and YFP-tagged EML4-ALK variants 1, 3, and 5 were kind gifts from Dr. Richard Bayliss. EGFRL858R was cloned into pBabe-puro and mTagBFP2-N1. iEGFR was constructed from full length EGFR (NM_005228.5) by PCR amplification using the following primers: ATGcgaaggcgccacatcgttcgg and gtgaatttattggagca. HOtag sequences for forced clustering were provided by Dr. Xiaokun Shu (Zhang et al., 2018). The RAS-GTP reporters (tandem GFP-RBD and GFP-RBD mutant) were a kind gift from Dr. Ignacio Rubio. cDNA sequence for GRB2 was cloned into the mEGFP-C1 vector. All mutants were generated through a combination of QuikChange site-directed mutagenesis (Agilent) and gene synthesis (Genewiz). The mutants contain the following modifications:

EML4-ALK full-length sequence GenBank: AB274722.1, using cDNA from bases 271 – 3450, mutations/deletions based on this sequence numbering. EML4-ALK kinase-deficient K589M: A2036T, EML4-ALK ΔHELP: deletion of bases 928-1158, EML4-ALK ΔTD: deletion of bases 310-459. CCDC6-RET full-length sequence GenBank: KU254649.1, cDNA from bases 1-1512, mutations/deletions based on this sequence numbering. CCDC6-RET kinase-deficient K147M: A440T, CCDC6-RET ΔCC: deletion of bases 160-303. EML4-RET was constructed using the N terminus of EML4 (bases 271-1759 of EML4-ALK sequence) fused to the RET kinase domain (bases 304-1512 of CCDC6-RET sequence). CCDC6-ALK was constructed using the N terminus of CCDC6 (bases 1-303 of CCDC6-RET) fused to ALK kinase domain (bases 1760-3450 of EML4-ALK).

#### DNA transfections

293T and Beas2B cells were transiently transfected using Mirus TransIt-LT1 transfection reagent according to manufacturer’s protocol.

#### Viral transduction

cDNAs for EML4-ALK, KRAS, HRAS, NRAS and respective cytosolic mutants were cloned into pLV-EF1a-IRES-blast (or hygromycin/puromycin selectable equivalent vectors). HEK293T viral packaging cells were plated in 10 cm dishes the day prior to transfection. They were transfected with lentiviral or retroviral expression constructs and the appropriate packaging plasmids using Mirus TransIt-LT1 transfection reagent. Viral supernatants were collected 48-72 hours post-transfection and used to transduce cell lines in the presence of 1 × Polybrene for 24 hours.

### Quantification and statistical analysis

#### Quantification of colocalization between EML4-ALK granules and endogenous signaling proteins

Customized MATLAB code was written to correct for uneven illumination pattern in the optical path and cell autofluorescence background. Granules were identified with Cellprofiler feature-finding module using Otsu thresholding method and size constraint from 0.4 to 2 μm in diameter. The pairwise centroid distance between features in BFP and GFP channels, corresponding to EML4-ALK granules and signaling proteins respectively, were calculated to identify colocalization events. Typically, ∼20 images containing 30+ cells and ∼300 granules for each signaling protein were analyzed in an automated batch-processing format. All colocalization events were confirmed manually by overlaying identified features with raw images.

#### Quantification of enrichment level of signaling proteins at EML4-ALK granules

Customized MATLAB code was written to identify pixels corresponding to EML4-ALK granules in the BFP channel in a given image. The intensity in the GFP channel at the positions of these pixels, corresponding to the enriched signaling proteins, was averaged for individual granules. The ratio of the average pixel intensity at the granule to the pixel intensity averaged over the whole cell area is the fold enrichment of the signaling protein at each granule.

#### Quantification of fraction of granule containing cells for EML4-ALK and CCDC6-RET mutants

Beas2B cells were seeded into Nunc Lab-Tek 8-well imaging chambers and plasmids encoding wild-type and mutant forms of mTagBFP2::EML4-ALK (i.e., ΔTD, ΔHELP, K589M, see text for details), mTagBFP2::CCDC6-RET (i.e., ΔCC, K147M), as well as YFP::EML4-ALK variants 1, 3, and 5 were transfected using Mirus TransIT-LT1. 24 hours later, an initial position in each well was randomly picked as the center of an area of 650 μm x 650 μm and imaged with automated scanning and tiling done through MicroManager with a 100x oil objective (N.A. = 1.40). The process was repeated three times and all the cells were scored manually to determine if they contained cytoplasmic granules.

#### Statistical analysis

*P value*s were determined with Student’s t tests or one-way ANOVA with post hoc Tukey’s HSD test between comparator groups using GraphPad software.
